# *Escherichia* Species Diversity Across Pristine and Impacted Catchments: Evidence for Avian Sources of *Escherichia marmotae* in Aotearoa/New Zealand

**DOI:** 10.1111/1758-2229.70278

**Published:** 2026-01-22

**Authors:** Adrian L. Cookson, Marie Moinet, Jonathan C. Marshall, Patrick J. Biggs, Lynn E. Rogers, Rose M. Collis, Megan Devane, Rebecca Stott, Richard Muirhead

**Affiliations:** ^1^ New Zealand Institute for Bioeconomy Science, AgResearch Group, Hopkirk Research Institute Massey University Palmerston North New Zealand; ^2^ mEpiLab, School of Veterinary Science, Massey University Palmerston North New Zealand; ^3^ School of Mathematics and Computational Sciences, Massey University Palmerston North New Zealand; ^4^ School of Food Technology and Natural Sciences, Massey University Palmerston North New Zealand; ^5^ New Zealand Food Safety Science and Research Centre, Massey University Palmerston North New Zealand; ^6^ New Zealand Institute for Public Health and Forensic Science Christchurch New Zealand; ^7^ Earth Sciences New Zealand Hamilton New Zealand; ^8^ New Zealand Institute for Bioeconomy Science, AgResearch Group, Invermay Agricultural Centre Mosgiel New Zealand

**Keywords:** ecosystem health, *Escherichia* spp., faecal sources, land‐use, public health, water quality

## Abstract

A total of 1347 *Escherichia* isolates from water, soil, sediment, biofilm, and faecal samples (*n* = 413) across five pristine (native forest) and five impacted (pastoral or urban) sites were subtyped into *E. coli* phylotypes and non‐*E. coli Escherichia* spp. ( *Escherichia marmotae* , *Escherichia ruysiae* and *Escherichia whittamii* ). Impacted sites showed a higher prevalence of *E. coli* , particularly the ruminant‐associated phylotype B1, across water, biofilm, sediment, and mammalian faeces. In contrast, *E. marmotae* (189 isolates) were more common in pristine sites and avian faeces, with a prevalence of 28.7%. Metabarcoding of the hypervariable gene *gnd* further revealed that *Escherichia* population diversity was greatest in aquatic environments (water, sediment, biofilm). *Escherichia* population diversity was also associated with elevated freshwater *E. coli* concentrations, increased prevalence of pathogenic *E. coli* virulence factors (*stx*1, *stx*2 and *eae*), and higher livestock numbers. In contrast, diversity measures for *Escherichia* populations were lowest in avian faeces and soil samples, and samples obtained from pristine sites with fewer faecal sources. These findings highlight the ecological role of birds as reservoirs of *E. marmotae* and their contribution to microbial diversity in New Zealand's freshwater ecosystems.

## Graphical abstract

*Escherichia* spp. were obtained from pristine and impacted environments to define community structure and assess potential human health risks. *E. coli* was more prevalent from sites affected by livestock or human activities, whereas non‐ *E. coli Escherichia* spp. were more prevalent in avian faeces and native forest with fewer faecal sources.

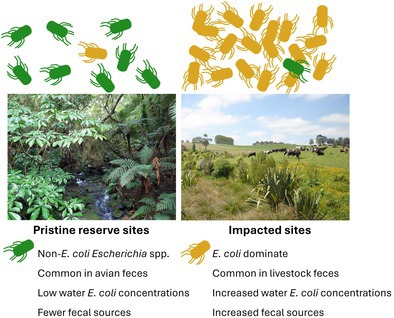

## 1 Introduction

*Escherichia coli* is a common inhabitant of the gastrointestinal tract of mammals and birds (Tenaillon et al. 2010; Blyton et al. 2015) yet it also frequently persists in environmental settings reflecting its resilience in extra‐intestinal habitats (Touchon et al. 2020). Consequently, waterborne contamination and human health risk are monitored using *E. coli* as an indicator of faecal sources and as a proxy for other microbial pathogens of faecal origin such as *Salmonella*, *Campylobacter*, *Cryptosporidium* and Shiga toxin‐producing *E. coli* (STEC) (Hörman et al. 2004; Till et al. 2008; Devane et al. 2020) that are more challenging in their culture and identification requirements. Thus, water bodies in areas with different observed land‐use impacts are often subject to regular *E. coli* testing using culture‐based methods to compare with, or inform the development of risk‐based guidelines to protect human health (Till et al. 2008). However, growing evidence challenges the long‐held view that *E. coli* is exclusively an indicator of recent faecal contamination. Studies have documented the persistence and apparent naturalisation of *E. coli* in environments considered pristine where direct inputs from human or livestock are minimal or absent (Tropea et al. 2021).

Urban freshwater sites, especially those impacted by point source discharge from wastewater treatment plants and pastoral sites with agricultural surface run‐off, typically show elevated *E. coli* concentrations that are of faecal origin (Cookson, Devane, et al. 2024). In contrast, native habitats with minimal human or livestock influence have lower *E. coli* loads, providing an opportunity to study environmental *E. coli* populations in settings with reduced faecal input (Petit et al. 2017; Cookson et al. 2022).

High resolution whole‐genome sequencing (WGS) methods have provided new insights into *E. coli* phylogeny, with subtyping identifying at least eight distinct phylotypes with characteristic hosts and ecological niches providing clues about likely faecal sources (Luo et al. 2011; Touchon et al. 2020). For example, phylotype B1 is commonly associated with the gastrointestinal tract of ruminants and is indicative of more recent faecal contamination events (Ishii et al. 2007; Johnson et al. 2017; Petit et al. 2017; Cookson, Marshall, et al. 2024). In contrast, phylotype B2 is more commonly isolated from extra‐intestinal habitats as a urinary tract pathogen (UTI) (Russo and Johnson 2000; Toombs‐Ruane et al. 2023) and found in forest habitats less impacted by diverse faecal sources (Petit et al. 2017; Cookson et al. 2022). Preliminary evidence has also suggested that increased freshwater *E. coli* concentrations are associated with greater *E. coli* diversity due to diffuse faecal contamination from livestock or urban areas (Cookson, Devane, et al. 2024).

Phylogenetic analysis of *E. coli* has also revealed the presence of cryptic clades (I to V) of *E. coli* ‐like bacteria that are indistinguishable by routine culture‐based or water quality assessment assays (Walk et al. 2009; Clermont et al. 2011) but readily separated using WGS data (Luo et al. 2011; Cookson et al. 2022). Clade I is generally regarded as *E. coli sensu stricto* due to its close genomic similarity to typical *E. coli* strains (Luo et al. 2011), whereas others have been assigned species designations: Clade II as *Escherichia whittamii* (Gilroy et al. 2021); Clades III and IV as *Escherichia ruysiae* (van der Putten et al. 2021); and Clade V as *Escherichia marmotae* (Liu et al. 2015). These non‐*E. coli Escherichia* species are occasionally isolated from clinical cases (Oladipo et al. 2025), often in immune‐compromised patients (Sivertsen et al. 2022; Chmel et al. 2025) and healthy individuals (van der Putten et al. 2021; Ho et al. 2025), and of low virulence (Ingle et al. 2011), but are primarily reported from environmental and wildlife sources (Blyton et al. 2015; Cookson et al. 2022; Sakaguchi et al. 2023; Moinet et al. 2024; Siddi et al. 2024; Liu et al. 2025; Poosakkannu et al. 2025). Preliminary studies suggest they are scarce in sites impacted by diverse faecal sources (Cookson, Devane, et al. 2024) and that their presence in birds reflects transient passage as a spillover host (Walk et al. 2009; Walk 2015) rather than gut colonisation. Although described as emerging human pathogens (Sivertsen et al. 2022; Oladipo et al. 2025), the environmental niche and origin of non‐*E. coli Escherichia* species remain uncertain, and they appear to be poor indicators of human health risk due to weak associations with human and livestock faecal sources. Their relevance as indicators of other pathogens in freshwater risk assessments, therefore, remains unclear (Sivertsen et al. 2022).

New Zealand (NZ) landscapes with their lack of endemic terrestrial mammalian species offer unique opportunities to examine environmental samples across sites with contrasting faecal sources and loadings. Bush reserves intensively managed to reduce introduced pests and predators in order to support populations of endemic species provide ideal sampling locations free of mammals. Pastoral and urban landscapes, however, represent sites impacted by comparatively higher faecal loads and multiple, often diffuse, faecal sources from animals and humans (Cookson, Devane, et al. 2024).

Our preliminary research identified non‐*E. coli Escherichia* species from a bush reserve undergoing intensive pest control (trapping) aimed at improving ecosystem health and supporting the survival of threatened endemic species (Cookson et al. 2022). Building on these findings, this site was chosen along with other pristine sites for a further paired‐site study where *Escherichia* spp. communities sampled from an intensively managed bush reserve site were compared with a corresponding downstream impacted site. The aim of this study was to investigate the sources and composition of non‐*E. coli Escherichia* species and to define the community structure of ‘natural’ *Escherichia* communities from pristine sites under relatively stable and undisturbed conditions. This was achieved using both culture‐based methods and metabarcoding of the hypervariable *gnd* gene (Cookson et al. 2017; Cookson et al. 2019; Cookson, Marshall, et al. 2024; Moinet et al. 2024) which encodes 6‐phosphogluconate dehydrogenase, an enzyme that catalyses the oxidative decarboxylation of 6‐phophogluconate as the final step of the pentose phosphate pathway. Specifically, we sought to determine whether non‐*E. coli Escherichia* species are more abundant in bush reserve sites with fewer faecal sources potentially serving as indicators of ecosystem health. These communities were contrasted with those from downstream sites impacted by pastoral and/or urban land use, where higher *E. coli* concentrations and increased potential human health risks are typically observed in waterways. We hypothesised that *E. marmotae* and related non‐ *E. coli* species would be more abundant in avian faeces and pristine sites, reflecting natural reservoirs rather than anthropogenic contamination.

## 2 Experimental Procedures

### 2.1 Sample Site Details

Ten field sites, comprising five paired locations (Figure S1), were sampled on six occasions during a 16‐month study (February 2020–June 2021), with each site pair sampled over a 12‐month period. Paired locations were selected to provide geographic coverage across NZ prioritising accessible reserve sites which allowed sampling activities and processing to occur within 24 h of collection. Three pairs were in the North Island of NZ and two in the South Island. The odd numbered sites (‘Reserve’ Sites01 to 09) were situated within pristine conservation areas that are intensively managed to eradicate introduced predatory pest species (mustelids, possums, hedgehogs and rodents) with the aim of enhancing native biodiversity particularly endemic bird species. Sites 03, 05, 07 and 09 were located within predator‐proof fence sanctuaries whereas Site01 remains unfenced but has ongoing and intensive pest trapping. The even numbered sites (‘Impacted’ Sites02 to 10) were located within pastoral farming areas (Sites02, 04, 08 and 10) and one in an urban setting (Site06). The linear distance between each reserve‐impacted site pair varied, ranging from 20.9 km (between Sites01 and 02) to 1.01 km (between Sites09 and 10).

### 2.2 Environmental Sampling and Bacterial Recovery

At each site: water, sediment, soil, aquatic biofilm (periphyton) (not Site03) and opportunistic avian and mammalian faecal samples were collected consistent with previous studies (Cookson et al. 2022; Cookson, Marshall, et al. 2024). *E. coli* were enumerated from water samples (100 mL) using Colilert‐18 and Quanti‐Tray/2000 (IDEXX, ME, USA) incubated at 35°C (18 to 21 h) for the recovery of stressed cells (Byappanahalli et al. 2006) to determine the most probable number of *E. coli* per 100 mL (MPN per 100 mL).

The recovery of individual isolates from environmental samples and DNA storage used methods described previously (Cookson et al. 2022; Cookson, Marshall, et al. 2024; Moinet et al. 2024) (Supporting Information). Broth culture (50 μL) from sample enrichments was inoculated onto CHROMagar ECC plates, streaked for individual colonies, and incubated at 35°C (18 to 21 h). For all sample types, four distinct colony isolates per sample enrichment were separately resuspended in EC broth containing glycerol (33% [w/v]) and stored at −80°C.

### 2.3 Molecular Analyses and Sub‐Typing of Isolates

A 284 bp partial *gnd* sequence was amplified by PCR (2*gnd*F and 2*gnd*R primers) and Sanger sequenced (ABI3730 DNA Analyzer, Massey Genome Service, Massey University, Palmerston North, NZ) as described previously (Cookson et al. 2017; Cookson et al. 2022; Moinet et al. 2024). The *gnd* sequence type (gST) from each *E. coli* isolate was identified using a custom‐made gndDb database of 766 distinct 284 bp *gnd* sequences obtained from genome sequence data (Cookson et al. 2019). *E. coli* phylotypes and non‐ *E. coli* species were identified using previously described PCR primers and conditions (Clermont et al. 2011; Clermont et al. 2013).

### 2.4 Identification of *stx1* , *stx2* and *eae* Virulence Factors Using Real‐Time PCR

Real‐time (RT‐) PCR was used to determine the prevalence of the diarrheagenic *E. coli* virulence factors responsible for the expression of Shiga toxin 1 (*stx*1), Shiga toxin 2 (*stx*2), and an epithelial cell adherence factor, intimin (*eae*). Environmental sample enrichments were examined for the presence/absence of each virulence factor using washed boiled lysates of post‐enrichment samples. RT‐PCR methods, primers, and probes have been described previously (Cookson et al. 2022). Culture enrichment samples and individual isolates were deemed positive for the RT‐PCR target with a *C*_*q*_ (quantification cycle) of < 35.

### 2.5 *Escherichia* spp. *gnd* Metabarcoding

For *Escherichia* community analysis, metabarcoding was used to amplify a 284 bp region of the *gnd* gene from the washed boiled lysates using indexed 2*gnd*F and 2*gnd*R PCR primers (Cookson et al. 2017; Cookson, Marshall, et al. 2024; Moinet et al. 2024). The individually indexed PCR products were pooled to create a mixture of libraries which were run on a single Illumina MiSeq (v.2 chemistry) lane with 2 × 250 bp paired‐end sequencing reads (Massey Genome Service, Massey University, Palmerston North, NZ). The *gnd* metabarcoding sequence reads were analysed using the DADA2 package (v.1.16.0) (Callahan et al. 2016) and the reads remaining post‐processing were used to construct an amplicon sequence variant (ASV) table. Individual ASVs (synonymous with gSTs) were mapped against the gndDb database (Cookson et al. 2019) to provide information on the *Escherichia* community profile associated with individual sample enrichments. A mock community positive control library was prepared with pooled equimolar amounts of separate purified PCR products with a unique combination of barcoded primers from seven different *E. coli* as described previously (Cookson et al. 2017; Cookson, Marshall, et al. 2024; Moinet et al. 2024).

### 2.6 Metabarcoding Data Analysis and *Escherichia* spp. Diversity

The ASV table, sample metadata and the matching gST table were combined to construct a phyloseq object using the R package Phyloseq v.1.32.0 (McMurdie and Holmes 2013). Chao1 and Shannon alpha diversity metrics, betadispersion (‘betadisper’) and rarefaction analysis (‘rarecurve’), were calculated using the vegan v.2.6–10 R package (Oksanen et al. 2020). Data visualisation was conducted in R v. 3.6.2 (R Development Core Team 2018) using a range of packages including ggpubr v.0.4.0 (Kassambara 2020) and ggplot2 v.3.5.2 (Wickham 2009). Significance levels of alpha diversity pairwise comparisons of *Escherichia* populations with Site or Sample type were adjusted for multiple testing using the Benjamini‐Hochberg false discovery rate (FDR < 0.05). Sankey plots were calculated using networkD3 v.0.4.1 (Allaire et al. 2025) where rare gSTs were removed by excluding triplet combinations of: Site, Sample, gST occurring ≤ 4 times. This threshold ensured inclusion of gSTs detected in at least two samples (i.e., representing five or more isolates) while excluding instances where four identical gSTs were identified within a single sample. Principal component analysis (PCA) of log_10_ transformed counts (log_10_(reads+1)) was used to identify population trends at the sample type and reserve/impacted site level.

### 2.7 Data Analysis Using Regression‐Based Models

Generalised linear mixed models (GLMMs), generalised linear models (GLMs), and linear mixed‐effects models (LMEs) were used to investigate associations between prevalence of bacterial characteristics (e.g., phylotype/ *Escherichia* spp.) and *stx*1, *stx*2, *eae* gene presence (i.e., whether a sample enrichment was positive using *C*_*q*_ < 35.0, or not) and environmental variables (e.g., sample type, land‐use, *E. coli* log_10_ MPN concentration etc.) at the sample and isolate level. GLMMs (binomial family with logit link function) were fitted using the glmer function from the lme4 package (v1.1–34) (Bates et al. 2015) in R to account for non‐independence among samples from the same catchment (impacted/Site pair), which was included as a random effect. These models were applied to sample‐level binary outcomes (e.g., presence/absence of a gene or phylotype) to obtain prevalence data. Alternatively, GLMs (binomial family) were used to investigate associations with site without any random effects. LMEs were fitted with the lme function from the nlme package (v.3.1–168) (Pinheiro and Bates 2025) for continuous outcomes where site was included as a random effect to investigate associations between *E. coli* log_10_ MPN concentration and land‐use, livestock numbers upstream of sample sites, catchment area (Ha), or linear distance (km) between each pair of sites. Shannon diversity and gST richness were also used as predictors in an LME model to evaluate their relationship with ‘distance’. Where appropriate, categorical predictors were used as references, with the reference level set to either a baseline sample type (e.g., avian faeces) or land use category (e.g., impacted) depending on the analysis.

### 2.8 Landscape Mapping and Catchment Characteristics

Land cover data was retrieved from the Land Cover Database v.5.0 (Anon 2020b), and riverine data from the River Environment Classification 2 (REC2) (Anon 2020a), using ipyleaflet within Jupyter and Python v.3.9 scripts. Ruminant livestock numbers (Anon 2021) were determined with grazing data from the Land Use and Carbon Analysis System (LUCAS) (Anon 2024) to calculate stocking densities according to land use.

The total area of site catchments ranged from 38.9 to approximately 19,000 Ha with catchment lengths of between 0.1 and over 86 km (Table S1). As a measure of total area, grassland ranged from approximately 7.16%–78.0% with impacted catchment livestock dominated by sheep (38.8%–96.0%). Although reserve sites were selected to be free of livestock within the sample site catchment, LUCAS data indicated limited livestock presence at Site 03 (*n* = 35; beef cattle and sheep reportedly present in an adjacent upstream paddock outside the reserve, based on local knowledge) and at Site 07 (*n* = 65; no livestock observed during sampling visits).

## 3 Results

### 3.1 Isolation of *Escherichia* spp. From Environmental Samples

Over the full 16‐month sampling period, a total of 60 site visits were conducted, with each of the 10 sites (5 reserve–impacted pairs) visited on 6 separate occasions, including bi‐monthly sampling over a 12‐month period. Samples were collected from the paired sites on the same day (e.g., Site01 and Site02). In total, 53 biofilm, 60 soil, 58 sediment, and 60 water samples were collected, with 182 opportunistic faecal samples (160 avian and 22 mammalian) (Table S2). Biofilm samples were not collected from Site03 as the riverbed substrate consisted of fine sands from which sediment samples were obtained. Generally, four separate presumptive *E. coli* isolates were recovered from each sample for subsequent analysis. No *Escherichia* spp. isolates were obtained from 3 biofilm, 8 sediment, 22 soil, 8 water, and 30 (29 avian, 1 cattle) faecal samples due to an absence of or poor microbial growth.

**FIGURE 1:**
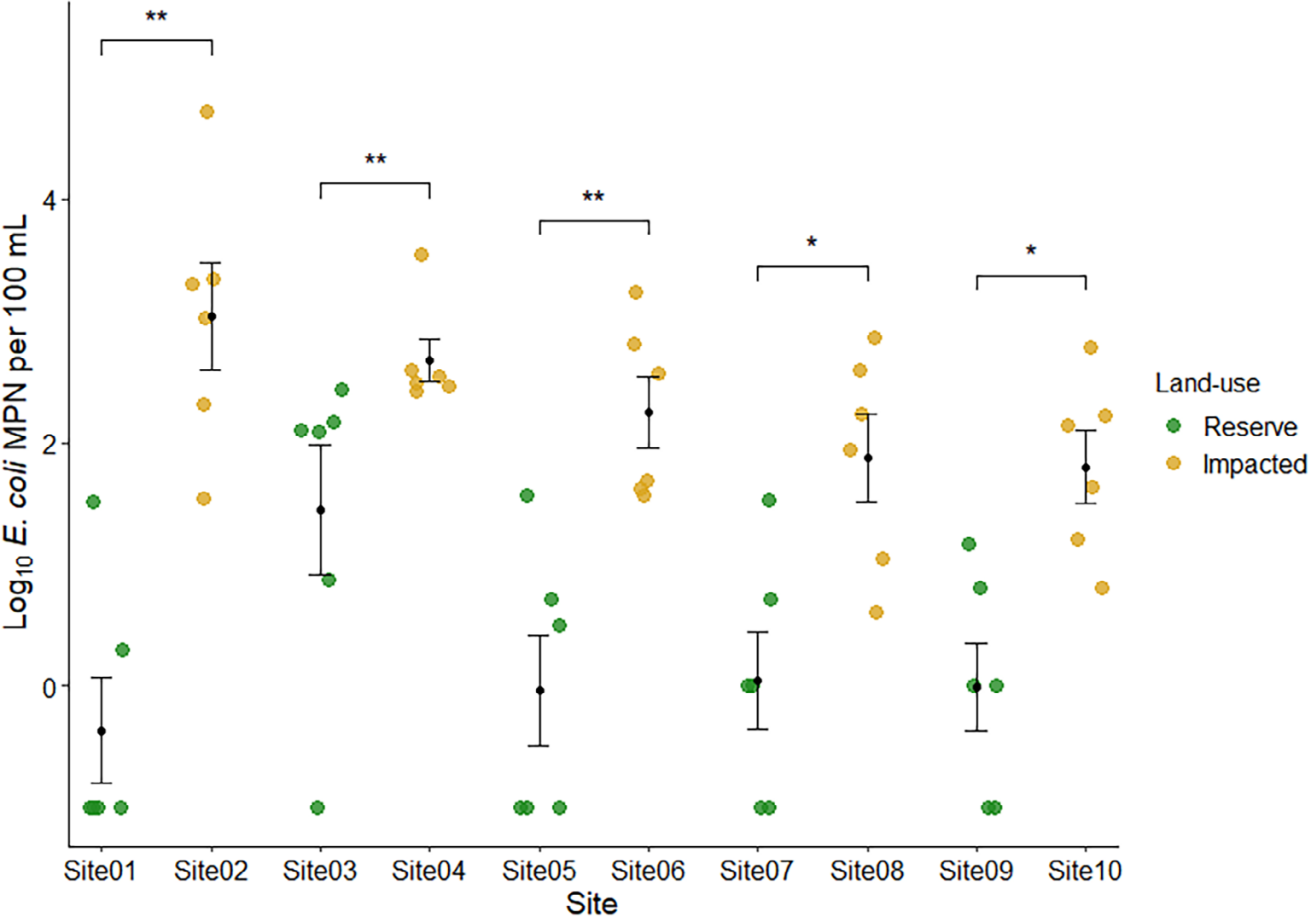
Prevalence of *E. coli* log_10_ MPN per 100 mL across sites. Points represent individual measurements from each site. Samples with 0 MPN per 100 mL were converted to 0.1 MPN before log transformation. Black points show the standard error for each site. Pairwise comparisons between adjacent sites in each pair (brackets) were performed using Wilcoxon rank‐sum tests, with significance indicated as: *p < 0.05, **p < 0.01.

*Escherichia* were isolated from all sites (Table S2) and most samples (342 of 413, 82.8%), including most biofilm (50 of 53, 94.3%), sediment (50 of 58, 86.2%), soil (38 of 60, 63.3%), water (52 of 60, 86.7%), and faecal samples (152 of 182, 83.5%) samples. Reserve sites accounted for most of the (63 of 71, 88.7%) samples from which no *E. coli* isolates were obtained. The geometric mean of freshwater *E. coli* counts ranged from 2 *E. coli* MPN per 100 mL (Site01, Pūkaha) to 1091 *E. coli* MPN per 100 mL (Site02, Hamua) with impacted sites having higher freshwater *E. coli* concentrations than their paired upstream reserve site (Figure 1).

### 3.2 Phylotyping and Prevalence of *Escherichia* spp. Across Sample Types and Land‐Use

Over the 60 sampling visits, 1351 presumptive *Escherichia* were isolated from the 10 sites. Four non‐*Escherichia* isolates (three *Hafnia* and one *Serratia*) were omitted from further analysis. PCR phylotyping was undertaken on the remaining 1347 isolates except when molecular subtyping to determine *gnd* sequence type (gST) (see below) was identical within a set of isolates from the same sample, for which phylotype was inferred from one isolate for up to three others. This approach was validated on a small subsample of 4% samples with 100% concordance in phylotype assignment. A phylotype could not be identified using conventional PCR for 24 isolates (1.78%); however, 22 isolates subsequently underwent WGS analysis and analysis of assembled genomes (Biggs et al. 2024) permitted the phylotype to be established. Phylotype B1 (37.7%, *n* = 508) and B2 (18.1%, *n* = 244) were the most common phylotypes identified, with other phylotypes including A, C, D, E, F, and G present at < 10%. Four cryptic clades were identified, including the *E. coli* ‐associated Clade I (0.07%, *n* = 1), Clade II/ *E. whittamii* (0.15%, *n* = 2), Clade III/IV/ *E. ruysiae* (0.74%, *n* = 10), and Clade V/ *E. marmotae* (21.4%, *n* = 289).

With avian faeces as the reference, the sample‐level prevalence of non‐*E. coli Escherichia* spp. was lower in sediment (OR 0.427, 95% CI: 0.20–0.916, *p* = 0.029) and soil (OR 0.471, 95% CI: 0.224–0.993, *p* = 0.048) but there was no difference compared to water (OR 1.028, 95% CI: 0.522–2.024, *p* = 0.937) (Table S3). Sample‐level prevalence of generic *E. coli* was also less in soil (OR 0.417, 95% CI: 0.204–0.854, *p* = 0.017), but greater in biofilm (OR 4.432, 95% CI: 1.74–11.27, *p* = 0.002), sediment (OR 2.33, 95% CI: 1.07–5.1, *p* = 0.034) and water samples (OR 4.15, 95% CI: 1.73–9.96, *p* = 0.001) compared with avian faeces.

**FIGURE 2:**
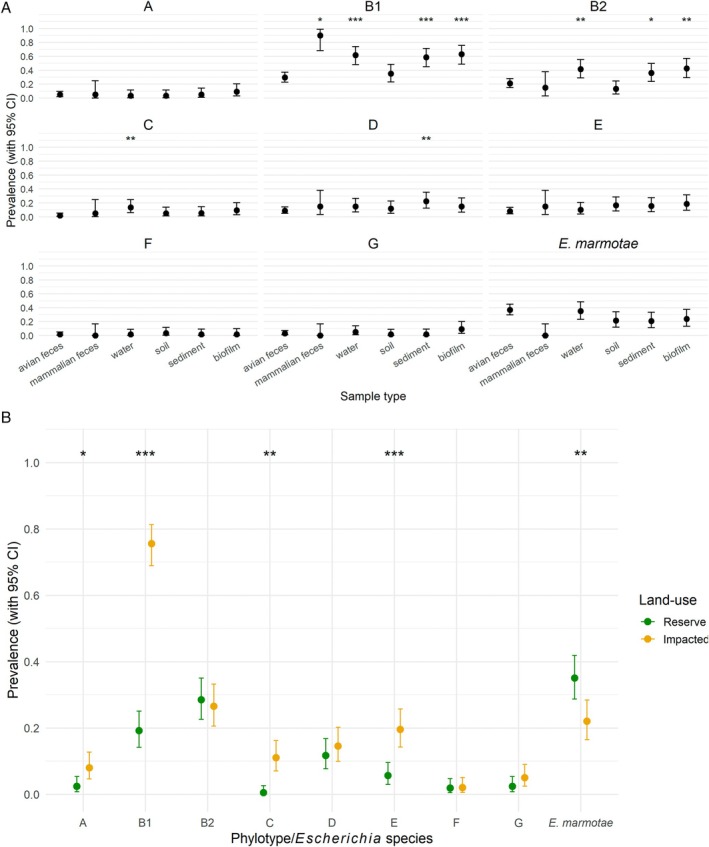
Prevalence of *E. coli* phylotypes and *E. marmotae* by (A) phylotype/species and (B) land‐use. Generalised linear mixed models were used to assess associations between the prevalence of *E. coli* phylotypes (A–G) and *E. marmotae* with sample type or land‐use. Where ‘sample type’ was used as a fixed effect, ‘avian faeces’ was included as the reference category and ‘Site’ was included as a random intercept to account for clustering of isolates within individual sites. Where ‘land‐use’ was used as a fixed effect, ‘impacted’ was included as the reference category and ‘land‐use/Site pair’ was included as a random intercept to account for clustering of isolates within individual sampling catchments. Mean prevalence for each phylotype is shown with error bars indicating 95% confidence intervals. The binary response variable indicated phylotype presence (1) or absence (0). Asterisks denote statistical significance of (A) phylotypes/species detection relative to avian faeces, and (B) reserve sites relative to impacted sites *p < 0.05; **p < 0.01; ***p < 0.001.

Sample types were diverse at the phylotype/ *Escherichia* spp. level, although no non‐*E. coli Escherichia* spp. were isolated from mammalian faeces (Figure S2A). Phylotype B1 was more prevalent from water, biofilm, sediment and mammalian faeces samples (*p* < 0.05) compared to avian faeces (Figure 2A). Phylotype C is most closely related phylogenetically to B1 (Touchon et al. 2020) and displayed similar environmental sample prevalence characteristics. For example, phylotype C was more commonly isolated from water samples compared to avian faeces, whereas phylotype D was more prevalent from sediment (*p* = 0.032) (Figure 2A). Compared with avian faeces, all other environmental sample types showed lower *E. marmotae* prevalence, but the differences were not statistically significant (*p* > 0.05) (Figure 2A).

When the sites were categorised into ‘reserve’ (Site01, Site03, Site05, Site07 and Site09) and ‘impacted’ (Site02, Site04, Site06, Site08 and Site10), reserve site samples were less likely to be positive for generic *E. coli* compared to impacted sites (OR 0.117, 95% CI: 0.055–0.251, *p* < 0.0001), but there was no difference between the overall association of non‐*E. coli Escherichia* spp. sample prevalence from ‘reserve’ and ‘impacted’ sites (OR 2.38, 95% CI: 0.734–7.738, *p* = 0.148).

Phylotypes B1 and B2, and *E. marmotae* were the dominant types isolated from each site (Figure 2B). At the individual site level B1 was the most prevalent phylotype from impacted sites (61%–100%) compared to *E. marmotae* which was the most prevalent *Escherichia* species from reserve sites (17%–65%), whereas phylotype B2 was equally prevalent across impacted (12%–43%) and reserve (23%–36%) sites (Table S4). When ‘land‐use’ was examined as a fixed effect with ‘impacted’ as the reference (Figure 2B), the prevalence of phylotype B1 was lower at ‘reserve’ sites (*p* < 0.001). There was no difference between the prevalence of phylotype B2 according to land‐use (*p* = 0.061), but phylotypes C and E were less prevalent from ‘reserve’ sites (*p* < 0.01). *E. marmotae* was more prevalent from the ‘reserve’ sites (*p* = 0.001). For the relationship between *E. coli* concentrations and phylotypes, as log_10_ MPN increased, only the odds of detecting phylogroup B1 increased (OR: 3.34, 95% CI: 1.67–6.71, *p* = 0.0006) (Table S5).

### 3.3 Molecular Subtyping of *Escherichia* spp. Using *gnd* Sequence Types

Bacterial subtyping of *Escherichia* isolates targeting a 284 bp fragment of the hypervariable *gnd* gene separated the 1347 isolates into 264 distinct *gnd* sequence types (gSTs) which were mapped against reference sequences contained in the gndDb reference database (Cookson et al. 2019). No PCR amplicon for Sanger sequencing was obtained from eight isolates but WGS allowed identification as gST041. The gST from two isolates could not be confirmed due to incomplete Sanger sequencing data and these two isolates were removed from subsequent gST analysis. Most gSTs (90.8%, 238 of 262) were associated with *E. coli* phylotypes (A to G) with the remaining 24 (9.2%) associated with non‐*E. coli Escherichia* spp. (22 *E. marmotae* , 1 *E. ruysiae* and 1 *E. whittamii* ). Of the non‐*E. coli Escherichia* spp. gSTs, most (*n* = 20) were specific to *E. marmotae* , *E. ruysiae* , or *E. whittamii* , whereas four (gST004, gST186, gST352, gST412) identified in PCR‐confirmed *E. marmotae* had previously been reported only from *E. coli* . Overall, gST514 (*n* = 46), gST258 (*n* = 45) and gST587 (*n* = 38) were isolated from the most samples and 83 gSTs (31.7%) were singletons only isolated once. On average 76 gSTs were isolated from the five impacted sites compared to 35 from the five reserve sites, with *E. coli* gSTs having a greater relative abundance from impacted sites compared to gSTs from non‐*E. coli Escherichia* spp. (Figure S3).

The link between the Site, sample type and gST parameters was represented in a Sankey plot where gSTs were examined at the *Escherichia* isolate level (Figure S4). Rare gSTs were removed by excluding triplet combinations of Site, sample type and gST with a frequency of four or less, excluding instances where the same four gSTs were identified within a single sample. The thickest shared flows were those involving avian faeces, spanning seven sites (including all reserve sites) and encompassing 15 gSTs. Five avian faeces gSTs were associated with non‐*E. coli Escherichia* spp. (four *E. marmotae* and one *E. ruysiae* ) (Figure S4). A further *E. marmotae* gST587 was represented in a flow involving water and biofilm sample types. Despite elevated *E. coli* contamination and gST diversity, impacted sites such as Site02 and Site04 were minimally represented, with only a single unique flow of gST497 from three horse faeces samples at Site02, and gST522 from soil samples at Site10.

### 3.4 Community Analysis of *Escherichia* spp. Using *gnd* Metabarcoding

In total 6.56 million reads from 326 samples (> 1000 reads) averaging 20,127 reads per library (maximum: 45,539 median: 25,593) were mapped against reference *gnd* sequences (gndDb). Barcoded *gnd* amplicons were obtained from samples recovered from all sites and most samples (365 of 413, 88.4%), including all biofilm (53 of 53, 100%) samples, and most faeces (160 of 182, 87.9%), sediment (52 of 58, 89.7%), soil (47 of 60, 78.3%) and water (53 of 60, 88.3%). A further 39 samples which generated less than 1000 reads after processing were removed leaving data from 326 samples for subsequent analysis. Overall, 1303 distinct ASVs were identified, and on average there were 29 ASVs per environmental sample (*n* = 326), with a maximum of 253 separate ASVs identified from one water sample (Site06). Rarefaction analysis performed across samples generated flattened curves indicating that the amplicon sequencing depth was sufficient to accurately estimate ASV richness and reflected that the observed diversity was close to the true diversity of the respective target environmental communities (Figure S5). By mapping ASVs against the gndDb reference database, ASVs were able to be separated into four groups (Table 1): ASVs which matched gSTs from the gndDb database; those ASVs which only matched non‐*E. coli Escherichia* species *gnd* sequences from gndDb; ASVs which matched both *E. coli* and non‐*E. coli Escherichia* species *gnd* sequences (gST004, gST186, gST352 and gST412) from gndDb; and remaining *gnd* ASVs from other *Enterobacteriaceae* with no match to gndDb. Most reads (72.9%) matched *E. coli gnd* sequences, 12.7% matched non‐*E. coli Escherichia* species, 2.5% both *E. coli* and non‐*E. coli Escherichia* species with the remaining 11.9% other *Enterobacteriaceae* (Table 1), but gSTs matching gndDb only formed 31.9% of all ASVs. All gSTs included in the mock community positive control libraries were detected in accordance with anticipated read numbers according to previous studies (Cookson et al. 2017; Cookson, Marshall, et al. 2024; Moinet et al. 2024).

**TABLE 1:** *Escherichia* community analysis using metabarcoding.

ASV group		Reads (%)	ASVs total (%)
1.	*E. coli* gSTs	4,785,003 (72.9)	387 (29.7)
2.	Non‐ *E. coli Escherichia* gSTs	835,778 (12.7)	25 (1.9)
3.	*E. coli* and non‐*E. coli Escherichia* gSTs	162,798 (2.5)	4 (0.31)
4.	Other *Enterobacteriaceae* ASVs	777,854 (11.9)	887 (68.1)
Total—all ASVs		6,561,433 (100)	1303 (100)

In the *Escherichia* community analysis using metabarcoding methods, the gST with the highest relative abundance at the read level was gST514 (4.04%, 265,349 reads) which was found in 38.0% samples (124 of 326, average 2140 reads) across all 10 sites, in faeces (avian, *n* = 43, mammalian, *n* = 7), water (*n* = 21) and biofilm (*n* = 20), sediment (*n* = 24) and soil (*n* = 9) and matched a gST from gndDb corresponding to *E. coli* . The second most abundant gST was gST587 (2.7%, 176,879 reads), a gST from gndDb corresponding to *E. marmotae* found in 24.2% samples (79 of 326, 2239 average reads) also across all 10 sites, in faeces (avian only, *n* = 16), water (*n* = 19), biofilm (*n* = 20), sediment (*n* = 17), and soil (*n* = 7).

Including gST587, 25 gSTs specifically representing 19 *E. marmotae* , 5 *E. ruysiae* (Clades III and IV) and a single *E. whittamii* (Clade II) were identified from *gnd* metabarcoding sequencing data. gST004, gST186, gST352, and gST412 were not included in this grouping as their exclusive provenance to non‐*E. coli Escherichia* species could not be confirmed. Overall non‐*E. coli Escherichia* species‐associated gSTs were identified from 67.5% (220 of 326) of samples, including faeces (avian only, 69.8%, 90 of 129), water (76.5%, 39 of 51), biofilm (76.9%, 30 of 39), sediment (84.0%, 42 of 50), and soil (41.3%, 19 of 46).

### 3.5 Alpha and Beta Diversity of *gnd* Sequence Types

**FIGURE 3:**
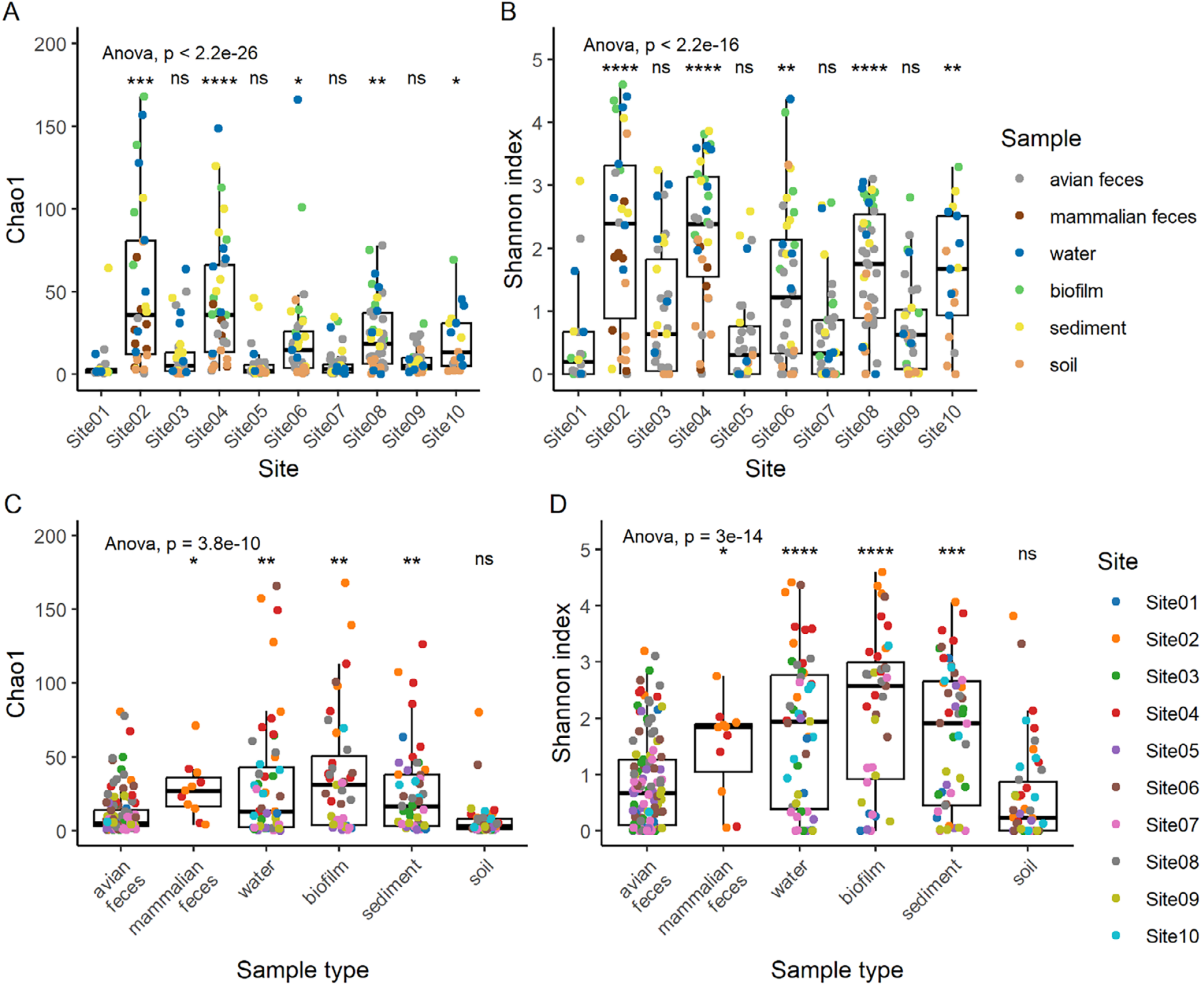
Alpha diversity analysis of *Escherichia coli* and *E. marmotae* , *E. ruysiae* and *E. whittamii* . Chao1 richness and Shannon diversity indices were calculated from metabarcoding data to assess variation in all *gnd* sequence types (gSTs; *n* = 416) matching *Escherichia* spp. within the *gnd* reference database (gndDb). Diversity profiles were derived from 326 environmental samples analysed by *gnd* amplicon metabarcoding. Amplicon sequence variants were identified using DADA2 and matched to gSTs in gndDb, which contains > 700 hypervariable partial *gnd* sequences from *Escherichia* species. Boxplots show median values (line), interquartile range (box), and whiskers extending to the most extreme values within 1.5× the interquartile range. (A, B) Student's *t*‐tests comparing Chao1 and Shannon indices from Site01 to each other site. (C, D) Student's *t*‐tests comparing Chao1 and Shannon indices from avian faeces to other environmental samples. Pairwise comparison *p* values were adjusted for multiple testing using the Benjamini–Hochberg false discovery rate (FDR), and only adjusted *p* values < 0.05 were considered significant. Significance codes: *p < 0.05; **p < 0.01; ***p < 0.001; ****p < 0.0001; ns = not significant (*p* > 0.05). A one‐way ANOVA was used to compare gSTs (*n* = 416) read counts across all sites and sample types.

Generally alpha diversity measurements of all *gnd* sequence types (gSTs) (*n* = 416) matching *E. coli* , and *Escherichia* species ( *E. marmotae* , *E. ruysiae* and *E. whittamii*) for impacted sites were significantly greater than Site01 (reserve reference site) (*p* < 0.05) (Figure 3A,B) with individual impacted site (Site02, Site04, Site06, Site08 and Site10) gST richness (Chao1) measurements increasing with greater linear distance between them (Figure 3A). Avian faeces and soil samples had decreased richness and within sample population diversity (Shannon indices) compared to water, biofilm, sediment and mammalian faeces (*p* < 0.01) (Figure 3C,D).

**FIGURE 4:**
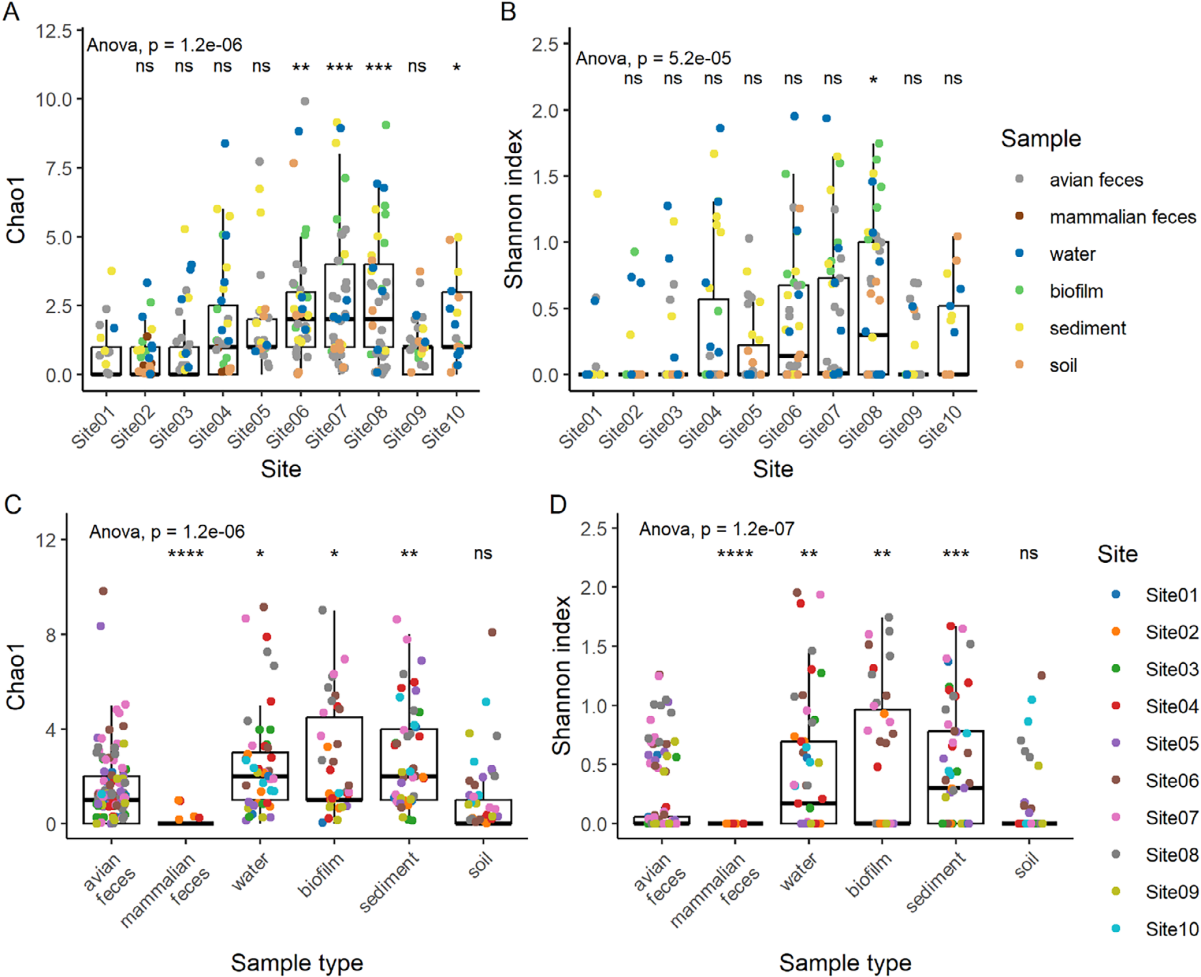
Alpha diversity analysis of *E. marmotae* , *E. ruysiae* and *E. whittamii* . Chao1 richness and Shannon diversity indices were calculated from metabarcoding data to assess variation in all *gnd* sequence types (gSTs; *n* = 25) matching only *E. marmotae* , *E. ruysiae* and *E. whittamii* within the *gnd* reference database (gndDb). Diversity profiles were derived from 326 environmental samples analysed by *gnd* amplicon metabarcoding. Amplicon sequence variants were identified using DADA2 and matched to gSTs in gndDb, which contains > 700 hypervariable partial *gnd* sequences from *Escherichia* species. Boxplots show median values (line), interquartile range (box), and whiskers extending to the most extreme values within 1.5× the interquartile range. (A, B) Student's *t*‐tests comparing Chao1 and Shannon indices from Site01 to each other site. (C, D) Student's *t*‐tests comparing Chao1 and Shannon indices from avian faeces to other environmental samples. Pairwise comparison *p* values were adjusted for multiple testing using the Benjamini–Hochberg false discovery rate (FDR), and only adjusted *p* values < 0.05 were considered significant. Significance codes: *p < 0.05; **p < 0.01; ***p < 0.001; ****p < 0.0001; ns = not significant (*p* > 0.05). A one‐way ANOVA was used to compare gST (*n* = 25) read counts across all sites and sample types.

The richness of gSTs corresponding to only non‐*E. coli Escherichia* species ( *E. marmotae* , *E. ruysiae* and *E. whittamii* ) (*n* = 25) was increased for Site06, Site07, Site08 and Site10 (*p* < 0.05) compared to Site01 (Figure 4A). Similarly, compared to Site01, there was an increased average Shannon diversity index for only Site08 (*p* < 0.05) (Figure 4B). Compared to avian faeces, there was a reduced richness (*p* < 0.0001) and Shannon diversity (*p* < 0.0001) of non‐*E. coli Escherichia* species gSTs in mammalian faeces (Figure 4C,D), indicating an increased gST diversity in water, biofilm and sediment ‘sink’ sources using data from metabarcoding detection methods.

**FIGURE 5:**
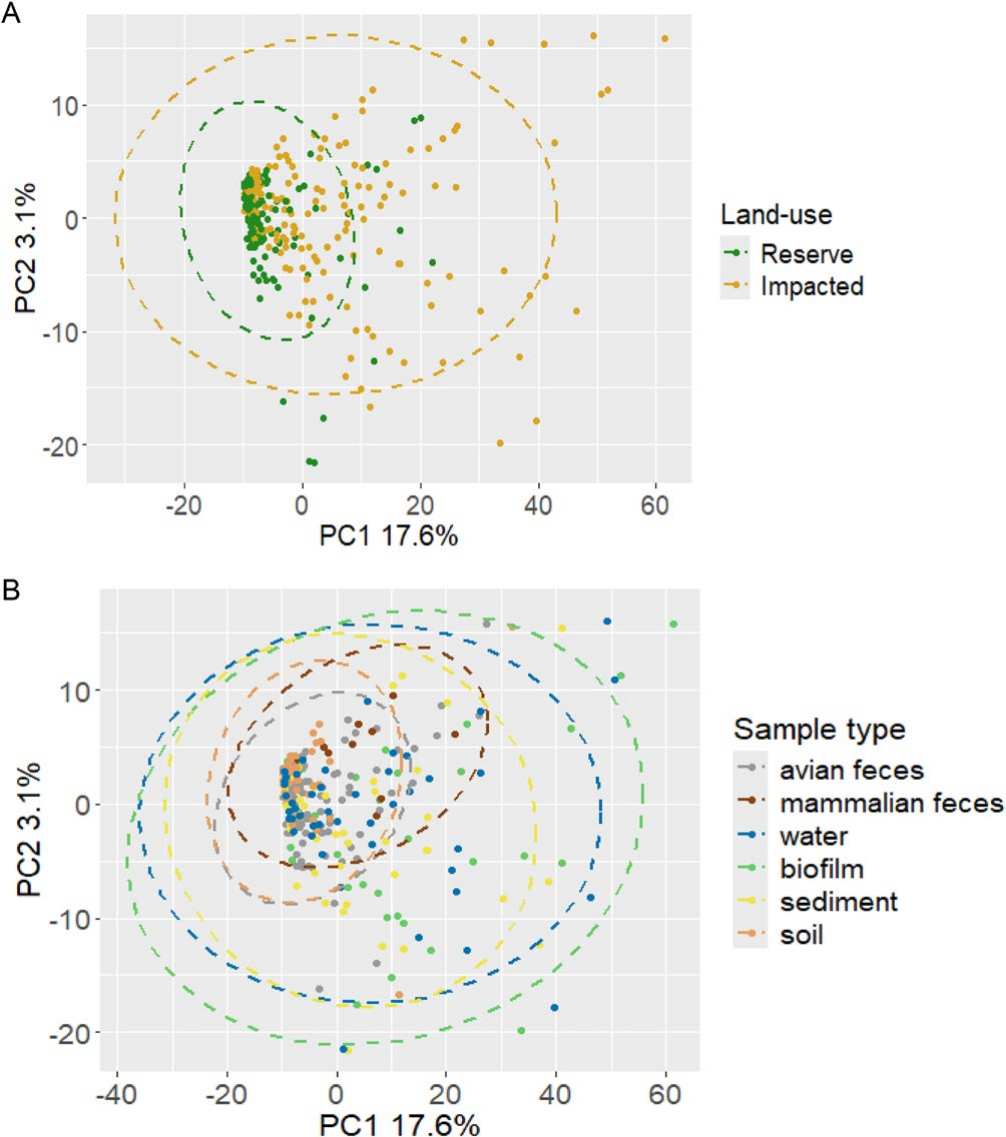
Beta diversity of *Escherichia coli* and non‐*E. coli Escherichia* species *gnd* sequence types (gSTs). Principal component analysis (PCA) illustrating variation and spatial distribution of gSTs (*n* = 416) matching *E. coli* , *E. marmotae* , *E. ruysiae* and *E. whittamii* in the *gnd* reference database (gndDb). Figures show differences by (A) land use and (B) sample type for environmental samples collected from 10 distinct sampling sites.

Subsequent PCA to examine between community diversity of the sample libraries constituting all *E. coli* and non‐*E. coli Escherichia* species gSTs (*n* = 416) from each respective library (*n* = 326) confirmed evidence of reduced beta diversity between sample *gnd*‐amplicon communities from the low‐impact reserve sites compared to corresponding paired sites downstream with pastoral or urban as the predominant land‐use class; group dispersions differed significantly (ANOVA on betadispersion distances, *F*(1, 324) = 6.17, *p* = 0.014) (Figure 5A). Similarly, between sample variation demonstrated that water, sediment and biofilm had increased beta diversity compared to avian and mammalian faeces, and soil; group dispersions differed significantly (ANOVA on betadispersion distances, *F*(5, 320) = 6.77, *p* < 0.0001) (Figure 5B).

Although only *E. coli* (*n* = 366) and non‐*E. coli Escherichia* species gST (*n* = 25) groupings (Table 1) were included in the alpha and beta diversity analysis (Figures 3 and 5), the inclusion of all ASVs, including those unassigned *Enterobacteriaceae* ASVs (Table 1) not matching gndDb, in subsequent analysis to demonstrate Shannon, Chao1, and PCA diversity of populations including all 1303 ASVs had minimal impact on the sample dispersion and overall clustering data (Figures S6A–F).

Linear mixed effects models indicated that *E. coli* concentrations (log_10_ MPN) were related to increased gST evenness (Shannon index, est: 0.368, 95% CI: 0.127–0.657, *p* = 0.009), but not gST richness (Chao1, est: 0.007, 95% CI: −0.0003 to 0.016, *p* = 0.081) of water samples (Table S6A). Similarly, lower *E. coli* log_10_ MPN concentrations were linked to Reserve sites (est: −2.11, 95% CI: −2.87 to −1.34, *p* = 0.0007) where Impacted was the reference (Table S6B) and for impacted sites only, *E. coli* log_10_ MPN tended to increase (est: 0.062, 95% CI: 0.024–0.099, *p* < 0.003) with linear distance downstream between the paired reserve and impacted sites (Table S6C). Also, as total number of livestock (divided by 100) upstream of the sampling location at impacted sites increased, *E. coli* log_10_ MPN also increased (est: 0.033, 95% CI: 0.0006–0.059, *p* = 0.047) (Table S6D); however, there was no significant interaction between log_10_ MPN and catchment area (1000 Ha units, est: 0.041, 95% CI: −0.001 to 0.085, *p* = 0.100) (Table S6E). Alpha diversity measures (richness, Chao1, est: 2.46, 95% CI: 0.027–4.63, *p* = 0.037; Shannon index, est: 0.067, 95% CI: 0.016–0.12, *p* = 0.014) relating to gSTs matching sequences from the gndDb reference database tended to increase with increased linear distance downstream (Table S6F).

### 3.6 Prevalence of Diarrhoeagenic *E. coli* Virulence Factors Across Sample Types and Land‐Use

Real‐time PCR (RT‐PCR) was undertaken to identify the presence of STEC‐associated genes (*stx*1, *stx*2 and *eae*) from 409 boiled lysate sample enrichments. *eae* was detected in 35.2% (144 of 409), *stx*2 in 23.7% (*n* = 97), and *stx*1 in 6.8% (*n* = 28) of DNA preparations (Table 2). Virulence genes were most commonly detected in mammalian faeces (*eae*, 59.1%; *stx*2, 59.1%; *stx*1, 31.8%) compared to soil (*eae*, 8.3%; *stx*2, 10.0%; *stx*1, 1.7%) (Table 2).

**TABLE 2:** Sample level prevalence of diarrhoeagenic *E. coli* virulence factors as determined using real‐time PCR.

Sample (*n*)	Virulence factor		
	*stx*1, *n* (%)	*stx*2, *n* (%)	*eae*, *n* (%)
Avian faeces (155)	3 (1.9%)	30 (19.3%)	44 (28.4%)
Mammal faeces (22)	7 (31.8%)	13 (59.1%)	13 (59.1%)
Biofilm (54)	5 (9.3%)	19 (35.2%)	33 (61.1%)
Sediment (58)	4 (6.9%)	21 (36.2%)	22 (37.9%)
Soil (60)	1 (1.7%)	6 (10.0%)	5 (8.3%)
Water (60)	8 (13.3%)	8 (13.3%)	27 (45.0%)

*Note:* Outcome positive samples were defined as gene presence *C*_*q*_ < 35.

**FIGURE 6:**
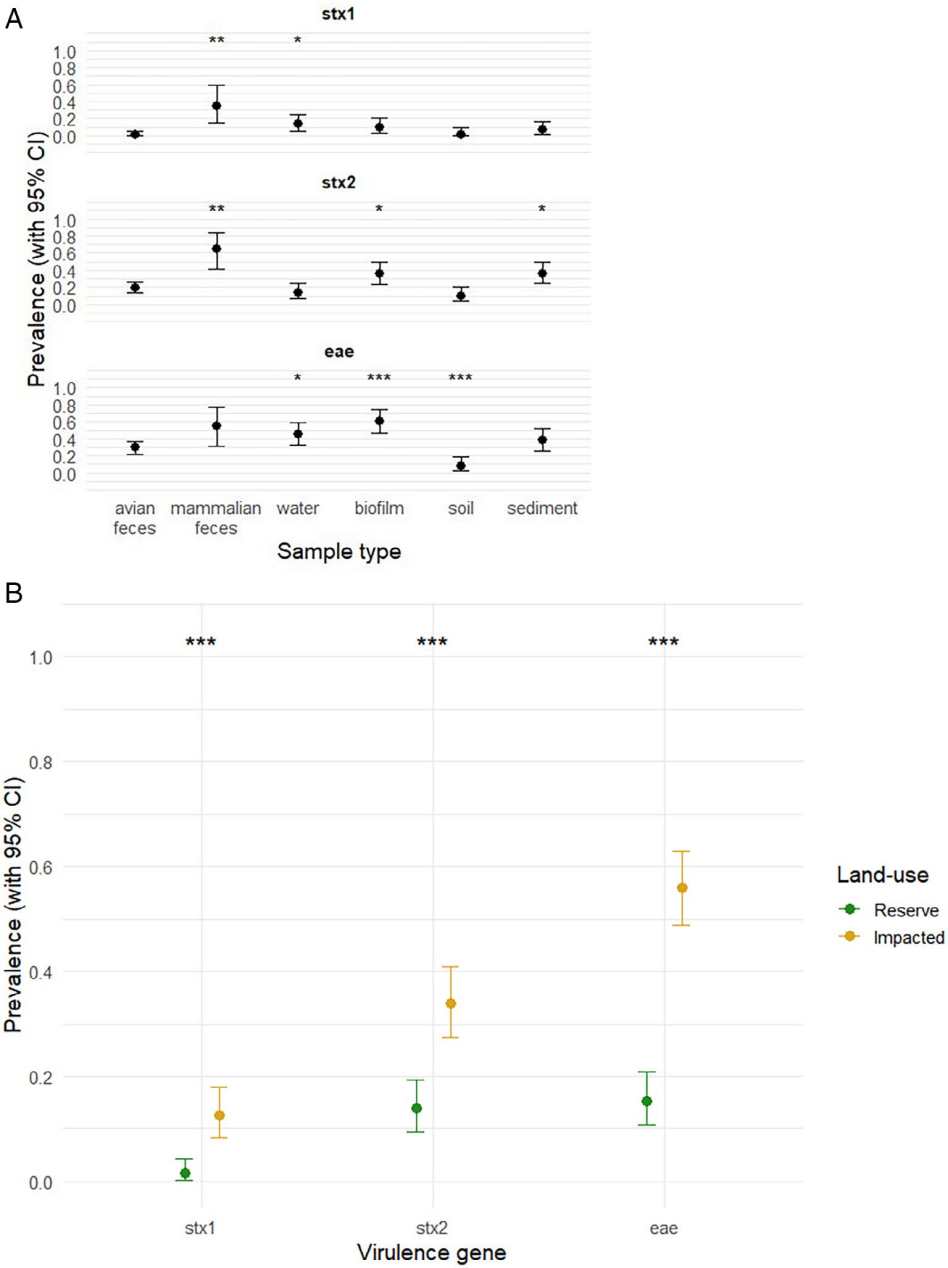
Prevalence of diarrhoeagenic *E. coli* virulence factors by (A) sample type and (B) land‐use. Generalised linear mixed models were used to assess associations between the prevalence of *stx*1, *stx*2, and *eae* with sample type or land‐use. Where ‘sample type’ was used as a fixed effect, ‘avian faeces’ was included as the reference category and ‘Site’ was included as a random intercept to account for clustering of isolates within individual sites. Where ‘land‐use’ was used as a fixed effect, ‘impacted’ was included as the reference category and ‘land‐use/site pair’ was included as a random intercept to account for clustering of isolates within individual sampling catchments. Mean prevalence for each virulence gene is shown with error bars indicating 95% confidence intervals. The binary response variable indicated virulence gene presence (1) or absence (0). Asterisks denote statistical significance of detection: (a) sample types relative to avian faeces, and (b) reserve sites relative to impacted sites (*p < 0.05; **p < 0.01; ***p < 0.001).

Generalised linear mixed effects models using RT‐PCR data to determine the presence or absence of *stx*1, *stx*2 and *eae* virulence factors indicated that *stx*1 was more prevalent in mammalian faeces (OR: 10.95, 95% CI: 2.07–58.01, *p* = 0.005) and water (OR: 6.21, 95% CI: 1.51–25.57, *p* = 0.012) compared to avian faeces (Figure 6A). *stx2* was more associated with mammalian faeces (OR: 4.63, 95% CI: 1.5–14.34, *p* = 0.008), biofilm (OR: 2.09, 95% CI: 1.01–4.32, *p* = 0.047) and sediment (OR: 2.26, 95% CI: 1.13–4.53, *p* = 0.021), and *eae* more prevalent in biofilm (OR: 5.1, 95% CI: 2.31–11.23, *p* < 0.0001) and water (OR: 2.09, 95% CI: 1.01–4.33, *p* = 0.047), but less prevalent in soil (OR: 0.13, 95% CI: 0.05–0.39, *p* < 0.0001). Compared to ‘impacted’ sites, all three virulence factors were much less prevalent in ‘reserve’ sites (*stx*1, OR: 0.09, 95% CI: 0.03–0.29, *p* = 0.005; *stx*2, OR: 0.29, 95% CI: 0.18–0.48, *p* = 0.0006; *eae*, OR: 0.13, 95% CI: 0.08–0.21, *p* < 0.0001) (Figure 6B).

*stx1* , *stx*2 and *eae* genes were also detected more often with increasing *E. coli* concentrations (log_10_ MPN) (*p* < 0.001) (Table 3A), with impacted sites (*p* < 0.01) (Table 3B) and alpha diversity measures (richness, *p* < 0.001; Shannon index, *p* < 0.001) relating to gSTs identified from metabarcoding data matching sequences from gndDb (Table S7). Phylotype B1 was more frequently detected in samples positive for *stx*1 (OR 17.34, 95% CI: 1.97–152.4, *p* = 0.01) and *eae* (OR 5.34, 95% CI: 3.0–9.53, *p* < 0.0001) (Table S8). However, phylotype B2 was negatively associated with *stx*2 (OR 0.52, 95% CI: 0.291–0.92, *p* = 0.025) (Table S8). There was no interaction between samples positive for *E. marmotae* and *stx*1, *stx*2 or *eae* genes.

**TABLE 3:** Odds ratios (OR) and 95% confidence intervals (CI) from binomial logistic models assessing associations between gene presence and environmental factors.

A					
Virulence gene	*E. coli* concentration	OR	95% CI	*z*	*p*
	Sample negative (reference)	1	Ref		
*stx*1	log_10_ MPN	6.58	1.60–27.026	2.61	0.009
*stx*2	log_10_ MPN	6.75	1.62–28.24	2.62	0.009
*eae*	log_10_ MPN	21.0	3.99–110.6	3.59	0.0003

B					
Virulence gene	Land‐use	OR	95% CI	*z*	*p*
	Impacted (reference)	1	Ref		
*stx*1	Reserve	0.089	0.017–0.48	−2.818	0.005
*stx*2	Reserve	0.294	0.146–0.593	−3.418	0.0006
*eae*	Reserve	0.134	0.063–0.285	−5.236	< 0.0001

*Note:* Results from generalised linear mixed models (GLMMs) examining the association between log‐transformed *E. coli* concentration (log_10_ MPN per 100 mL) and the presence of *stx1*, *stx2* or *eae* genes in sample enrichments (A). Models were fitted with a logit link, including gene presence (*C*_*q*_ < 35, 1) or absence (*C*_*q*_ > 35, 0) as the binary outcome variable. Log‐transformed *E. coli* concentration was included as a fixed effect, and site was included as a random intercept to account for clustering of samples within sites. Odds ratios represent the change in odds of gene detection per unit increase in log_10_ *E. coli* concentration. Results from generalised linear models (GLMs) assessing the association between land‐use and the presence of *stx1*, *stx2* or *eae* genes in sample enrichments (B). Separate models were fitted for each gene using a logit link. Land‐use was included as a fixed effect, with ‘impacted’ sites designated as the reference category. Odds ratios represent the relative odds of gene detection at ‘Reserve’ sites compared to the reference sites.

## 4 Discussion

The aim of this study was to combine culture‐based and metabarcoding approaches to characterise non‐*E. coli Escherichia* communities and their potential sources across contrasting land‐use settings. The study sought to determine whether these species are more prevalent in low‐impact bush reserves with minimal faecal inputs and thus may serve as indicators of ecosystem health relative to downstream sites influenced by urban and/or pastoral activity. Additionally, to assess their potential as a source and explore connectivity between birds and the environment, more avian faecal samples were collected and examined than sink samples (e.g., water, soil, sediment, biofilm), reflecting the opportunistic nature of faecal sampling and the proportionally greater occurrence of bird faeces observed in bush reserves during site visits.

*E. coli* is commonly used as an indicator of faecal contamination (Devane et al. 2020) as it is a common inhabitant of the intestine of mammals and birds (Touchon et al. 2020). This premise was explored in detail in this study by sampling relatively stable and undisturbed sites with high endemic biodiversity paired with downstream sites associated with pastoral and urban activity to understand potential faecal sources and human health risk. The scarcity of terrestrial mammalian species endemic to NZ provides a unique opportunity to undertake comparisons of *Escherichia* community structure with an emphasis on *E. coli* as a faecal indicator species from sites of contrasting land use. The reserve sites in this study were specifically chosen as they represent pristine forest environments where introduced pests and predators are carefully managed with negligible faecal sources from mammalian species including livestock.

Our previous work at Site01 (Pūkaha Mount Bruce) (Cookson et al. 2022), which was used as our logistic regression model reference site, demonstrated reduced freshwater *E. coli* concentrations compared to impacted downstream sites. *E. coli* concentrations were also lower in freshwater samples taken from ‘low impact’ sites consisting of catchments dominated by native and exotic forest in another recent study (Cookson, Devane, et al. 2024). This trend of low *E. coli* concentrations from freshwater samples obtained in reserve sites was replicated in the current study. However, even relatively small numbers of livestock in upper catchment sites characterised by open grassland for pastoral grazing (e.g., Site03) were associated with a one order of magnitude increase in *E. coli* log_10_ MPN, an increased prevalence of the ruminant‐associated phylotype B1 (Ishii et al. 2007; Johnson et al. 2017; Petit et al. 2017), and a low *E. marmotae* prevalence (20%) compared to an average of 37% for the other reserve sites. These data, together with the increased alpha diversity of gSTs matching gndDb observed at Site03 driven by some water samples (Figure 3B), clearly indicate increased faecal sources and higher levels of phylotype B1 from livestock contamination originating upstream of the fenced reserve. In contrast, there was no increase in *E. coli* log_10_ MPN, phylotype B1, or alpha diversity measures from Site07, where mapping data indicated the presence of livestock upstream external to the reserve but where none were observed.

Notably Site06 was the only urban site with minimal upstream livestock influence and showed a low level of phylotype B1 (61%) but a high prevalence of *E. marmotae* (48%). These data suggest that in the absence of major livestock inputs, birds may represent an important and consistent faecal source rather than being incidental carriers. Similarly, at impacted Site08, located only 500 m from the reserve perimeter, elevated *E. marmotae* prevalence indicates that avian faecal inputs predominated under reduced livestock influence, supporting birds as a source of *E. marmotae* dissemination in the environment.

Phylotype B2, often associated with urinary tract infections (UTIs) and bacteraemia (Russo and Johnson 2000; Toombs‐Ruane et al. 2023), was detected across both pristine and impacted sites. Previous studies have linked phylotype B2 to low‐impact environments (Petit et al. 2017; Cookson et al. 2022; Cookson, Devane, et al. 2024), however, an increased prevalence of B2 was observed at some impacted sites located close to pristine areas, suggesting potential environmental connectivity or dispersal from upstream sources. Further genomic studies are needed to determine whether environmental B2 lineages resemble those implicated in extra‐intestinal infections, and to assess whether the absence of specific virulence factors may influence their pathogenicity in humans (Smati et al. 2015). Along with phylogroup B2, phylogroups D and F comprise the majority of the extra‐intestinal pathogenic *E. coli* strains (Escobar‐Paramo et al. 2004). Phylotype E is a less common *E. coli* lineage displaying considerable genetic diversity with a population structure mirroring that of the whole species (Clermont et al. 2021), and compared to impacted sites, it was less associated with pristine reserve sites, indicating a possible livestock source (Figure 2B).

The potential role of non‐*E. coli Escherichia* spp. as opportunistic pathogens remains uncertain. Several studies have demonstrated the isolation of *E. marmotae* from bloodstream infections in patients with underlying conditions (Sivertsen et al. 2022; Chmel et al. 2025), but mouse colonisation studies provided no indication of virulence (Ingle et al. 2011). Faecal isolates have also been obtained from humans with no underlying conditions indicative of a commensal lifestyle (van der Putten et al. 2021; Ho et al. 2025). Mounting evidence, however, suggests that non‐*E. coli Escherichia* spp. may acquire plasmids containing antimicrobial resistance genes, which, coupled with plasmid‐associated virulence genes (Alonso et al. 2018; Campos‐Madueno et al. 2023; Sakaguchi et al. 2023; Siddi et al. 2024; Chmel et al. 2025), may enhance pathogenicity and progression to an emerging pathogen. WGS data (Biggs et al. 2024) may provide clues on potential horizontal gene transfer events and whether non‐*E. coli Escherichia* spp. isolates recovered from impacted sites with diverse faecal sources can be phylogenetically distinguished from those isolated from pristine reserve sites through identification of acquired antimicrobial resistance genes or *E. coli* virulence factors.

Alongside identifying virulence factors, characterising the ecological habitats of non‐*E. coli Escherichia* spp. is crucial for waterborne risk assessments, to assess their direct risk to human health, and to evaluate their utility as indicators of faecal contamination and indirect health risks. The isolation of non‐*E. coli Escherichia* spp. from wildlife (Blyton et al. 2015; Liu et al. 2015; Zhurilov et al. 2023; Siddi et al. 2024; Liu et al. 2025) and environmental sources (Vignaroli et al. 2015; Koh et al. 2022; Mire et al. 2022; Moinet et al. 2024) is well established, but less so for extensive livestock systems (Ocejo et al. 2020) more routinely associated with increased risk from zoonotic pathogens such as STEC, *Campylobacter*, *Salmonella* and *Cryptosporidium* (Phiri et al. 2020). Findings from this study suggest that for NZ at least, *E. marmotae* may be more prevalent in habitats containing few faecal sources such as native forest ecosystems with high levels of endemic biodiversity. This highlights its potential as a microbial indicator of ecosystem health in low‐impact conservation‐managed landscapes.

Statistical models applied in this study revealed direct links between multiple parameters associated with increased faecal‐source diversity and the prevalence of non‐*E. coli Escherichia* spp. Impacted sites exhibited higher *E. coli* concentrations alongside higher Shannon diversity of *Escherichia* gSTs, consistent with increased source mixing and improved detection that elevates evenness. In contrast, reserves with low MPN were often dominated by fewer lineages, particularly non‐*E. coli Escherichia*, resulting in lower Shannon diversity. Compared to reserve sites, impacted sites had higher *Escherichia* diversity, elevated *E. coli* concentrations, and greater livestock presence, patterns that were generally associated with increased downstream distance from the paired reserve site. Livestock, including cattle and sheep, are the main reservoir of diarrhoeagenic *E. coli* virulence factors *stx*1, *stx*2 and *eae* (Cookson et al. 2006; Jaros et al. 2016; Browne et al. 2018) and a suggested source for human infection either through direct contact or exposure to environments contaminated with ruminant faeces (Jaros et al. 2013). The *stx*2 variant is more commonly associated with STEC from cattle compared to *stx*1 (Cookson et al. 2006) and concordant with previous studies (Cookson et al. 2022) was more likely to be detected from sites impacted by livestock faecal sources. Indeed, all three virulence factors were more prevalent in samples from impacted sites and samples with increased alpha diversity indicative of diverse faecal sources.

NZ studies thus far have identified several *Escherichia* spp. cryptic clades. While *E. marmotae* has been isolated on multiple occasions, *E. ruysiae* has been detected much less frequently (Cookson et al. 2022; Cookson, Devane, et al. 2024; Moinet et al. 2024) and this study is the first to report *E. whittamii* in NZ. Non‐*E. coli Escherichia* spp., including *E. marmotae* and *E. ruysiae*, have been identified in previous NZ studies with an isolate‐level prevalence of < 10% (Cookson et al. 2022; Cookson, Devane, et al. 2024). However, in this study, compared to paired reserve and pristine sites, non‐*E. coli Escherichia* spp. represented 22.3% of all *Escherichia* isolates (*n* = 1457). Among these, *E. marmotae* (21.4%) was still more frequently identified than *E. ruysiae* (0.74%) and *E. whittamii* (0.15%). The specific hosts and/or environmental factors influencing this contrasting prevalence remain unclear.

Non‐*E. coli Escherichia* spp., especially *E. marmotae* , were most frequently isolated from avian faeces sampled from reserve sites indicating distinct ecologies compared to generic *E. coli* . However, whether this preponderance of *E. marmotae* in avian hosts is influenced by individual host specificity, enhanced environmental persistence, or reduced competition with *E. coli* from more diverse faecal sources associated with impacted sites requires further investigation. Initial phylogenetic analysis of disparate *E. marmotae* misclassified as *E. coli* from published genome assemblies suggested an undefined population structure independent of ecological niche (Binsker et al. 2024). Nevertheless, data from this study including the high prevalence of *E. marmotae* provide strong evidence that bird species endemic to NZ could be natural hosts of *E. marmotae* rather than a spillover or a transitory host acquiring bacteria from the environment as previously postulated (Walk 2015).

Using metabarcoding methods, there was less *Escherichia* spp. diversity in avian faecal samples and soil compared to extra‐intestinal aquatic environmental niche types sampled in this study (water, sediment, biofilm) (Figures 3C and 4C). This lower overall diversity of *Escherichia* spp. may have aided the higher frequency of non‐*E. coli Escherichia* species isolated from avian faeces, especially if birds serve as preferred hosts. Furthermore, Sankey plots illustrating unique triplet combinations of Site, Sample, gST with frequencies of > 4 revealed avian faeces as a key conduit and often leading to *E. marmotae* gSTs.

Previous studies have demonstrated that the adaptation of *Escherichia* spp. to the gut environment can be driven by periodic selection and clonal interference mediated by nutrient competition and host immune responses (Chang et al. 2004; Barroso‐Batista et al. 2014; Gordo et al. 2014; Massot et al. 2017). In zoonotic pathogens, such adaptations occur as mutations, and although point mutations occur more frequently, it is recombination events which provide a significant factor in structuring populations (Guttman and Dykhuizen 1994; Biggs et al. 2011). These selective pressures benefit a few well‐adapted lineages through purifying selection limiting diversity and maintaining dominant strains best suited to the gut niche. In contrast, biofilm, sediment, and water function as microbial sink environments, harbouring strains from a wide range of faecal sources. This mixing of inputs fuels greater environmental diversity than the selective bottlenecks imposed by a single host gut, as seen in the avian faeces data.

The O‐antigen biosynthesis gene cluster (O‐ABGC) encoding the *E. coli* lipopolysaccharide moiety is a hotspot for recombination in *E. coli* driving antigenically distinct O serogroups (Guttman and Dykhuizen 1994; Milkman et al. 2003). The *gnd* gene encoding the 6‐phosphogluconate dehydrogenase enzyme is positioned close to the O‐ABGC and is itself impacted by recombination (Nelson and Selander 1994; Gilmour et al. 2007), and is thus an ideal target for high resolution subtyping of purified isolates and metabarcoding for *Escherichia* spp. population analysis through identification of gSTs (Cookson et al. 2017) assigned using the custom‐built database of *gnd* amplicons (Cookson et al. 2019). However, although a potential limitation of this *Escherichia* subtyping method is that sample enrichments are required to facilitate *Escherichia* spp. isolation and also for *gnd* amplification as part of the metabarcoding community analysis protocol, the enrichment step is especially pertinent when *Escherichia* spp. may be present at low concentrations from environmental samples. The same sample enrichments were used for both isolate purification and metabarcoding, with gST514 emerging as the most abundant gST in metabarcoding and also the most frequently isolated gST. This strong concordance between methods supports and validates the use of *gnd*‐based subtyping where the same reference database gndDb can be used for subtyping isolates as well as for classifying ASVs as gSTs with metabarcoding methods. Although the enrichment step is a limitation of the metabarcoding method concealing the relative abundance of *Escherichia* spp. in their native state, it is necessary to increase biomass of targeted microbial species and reduce the likelihood of DNA amplification from non‐viable bacteria.

Unequal sampling across avian and mammalian hosts limits direct comparison; nevertheless, further genomic analysis of *E. marmotae* may help confirm the functional basis for the distinct patterns observed between pristine and impacted sites and among host types. Other experimental limitations include the use of the hypervariable *gnd* gene as a single marker. Unlike our previous studies where non‐*E. coli Escherichia* spp. have been identified with unique gSTs (Cookson et al. 2022; Moinet et al. 2024), here four new gSTs associated with *E. marmotae* matched those previously associated with *E. coli* and that had already been included in the gndDb reference database. For gST identification only a 284 bp fragment of the complete 1407 bp *gnd* gene is utilised for designation and inclusion in gndDb (Cookson et al. 2017); hence although the 284 bp amplicons from gST004, gST186, gST352 and gST412 from *E. coli* and *E. marmotae* are identical, future studies to examine the full length *gnd* gene may identify allelic variation and be species‐specific. Alignment searches using the BLAST database indicated no previous matches with *E. marmotae* submitted to GenBank for three of the shared gSTs (gST186, gST352 and gST412), but two *E. marmotae* matches (CP072689, CP146380) were identified for the remaining gST (gST004). Furthermore, *gnd* is also found in many other Enterobacterales which unlike *E. coli* , may not be indicators of faecal contamination (Cookson et al. 2017; Cookson, Marshall, et al. 2024; Moinet et al. 2024). This broad taxonomic distribution of the *gnd* allele consistently gives rise to the identification of many ASVs which do not match gSTs from gndDb. However, as these non‐assigned ASVs are often associated with low read numbers, their overall impact on diversity measures is limited. Alignment investigations of unassigned ASVs do nonetheless provide a resource for the identification of hitherto unknown gSTs from *E. coli* and non‐*E. coli Escherichia* spp. for inclusion in gndDb (Cookson et al. 2017).

This is currently the most comprehensive study to identify potential sources and hosts of non‐*E. coli Escherichia* spp. and to unravel the community diversity of *Escherichia* spp. from environmental samples across contrasting land‐uses. Findings point to distinct habitats for *E. marmotae* and *E. coli* , with avian species as a likely host of *E. marmotae* in NZ bush environments. The consistent detection of non‐*E. coli Escherichia* species, especially *E. marmotae* , in bird faeces across sites with low inputs from livestock faeces supports the inference of birds as a primary reservoir and likely source of environmental dissemination, rather than passive recipients from environmental exposure. Furthermore, by identifying non‐*E. coli Escherichia* species as potential markers of low impacted sites and ecological integrity, this research contributes to the development of microbial indicators for freshwater ecosystem health. Importantly, their reduced prevalence in downstream, fecally contaminated sites, where they are masked by numerically dominant generic *E. coli* from diverse faecal sources, provides assurance to water managers that non‐*E. coli Escherichia* spp. are unlikely to compromise water quality assessments for public health risk. Moreover, their presence may complement existing monitoring frameworks by indicating reduced faecal contamination and overall ecosystem health, ultimately supporting more targeted conservation strategies. It would be valuable however, to conduct a parallel study overseas to assess the influence of endemic mammals as sources of faecal contamination in freshwater and their impact on the prevalence of non‐*E. coli Escherichia* species in forest environments abroad. Future studies integrating whole‐genome and functional analyses are warranted to determine whether *E. marmotae* forms stable associations with avian hosts, to elucidate its pathogenic potential, and to assess its value as an indicator of freshwater ecosystem integrity.

## Author Contributions

**Adrian L. Cookson:** conceptualization, methodology, validation, formal analysis, investigation, data curation, writing – original draft, visualization, supervision, project administration, funding acquisition. **Marie Moinet:** methodology, validation, investigation, resources, data curation, writing – review and editing. **Jonathan C. Marshall:** conceptualization, investigation, methodology, software, validation, formal analysis, resources, data curation, writing – review and editing. **Patrick J. Biggs:** conceptualization, methodology, investigation, software, validation, formal analysis, resources, data curation, writing – review and editing. **Lynn E. Rogers:** investigation, writing – review and editing. **Rose M. Collis:** investigation, writing – review and editing. **Megan Devane:** conceptualization, methodology, investigation, writing – review and editing. **Rebecca Stott:** conceptualization, methodology, investigation, writing – review and editing. **Richard Muirhead:** conceptualization, methodology, investigation, writing – review and editing.

## Funding

This work was supported by the New Zealand Ministry of Business, Innovation and Employment through Smart Idea project C10X1908 and through AgResearch Strategic Science and Investment funding.

## Conflicts of Interest

The authors declare no conflicts of interest.

## Supporting information


**Figure S1:** Catchment map of sampling sites. Five paired sites consisted of an upstream headwater site (odd‐numbers, shown in blue) located within a pristine native bush reserve, and a corresponding downstream site (even‐numbers, shown in orange) impacted by pastoral or urban land use.
**Table S1:** New Zealand land‐use and livestock data details associated with each sampling site. ^a^ No water was detected in these catchments as extensive native forest cover precludes satellite detection of lower order streams. ^b^ refers to the number of livestock animals in the upstream catchment area.
**Table S2:** New Zealand sample site details where water, soil, sediment, biofilm (periphyton) and faecal samples were obtained for this study. ^a^ Linear distance between paired sites; ^b^ number of samples where *Escherichia* isolated; ^c^ not collected, sandy riverbed.
**Table S3:** Prevalence of *E. coli* phylotypes and non‐*E. coli Escherichia* species from the 10 sample sites. The odd‐numbered sites refer to those within native bush reserves and the even‐numbered sites refer to those within impacted sites and more heavily influenced by diffuse faecal sources. Sites are separated into individual Site pairs contained within the same freshwater catchment.
**Table S4:** Odds ratios (OR) and 95% confidence intervals (CI) from binomial generalised linear mixed models (GLMMs) assessing the association between sample type and the generic *E. coli* (phylotypes A to G) and non‐*E. coli Escherichia* ( *E. marmotae* , *E. ruysiae* and *E. whittamii* ). Models were fitted using a logit link function, with the binary outcome variable indicating presence or absence of response variable bacteria from individual sample enrichments. Sample type was included as a fixed effect, and site was included as a random intercept to account for clustering. Odds ratios represent the change in odds of an isolate being specified as a particular phylotype relative to the reference avian faeces sample type.
**Figure S2:** Relative abundance (%) of *E. coli* phylotypes (A to G) and *E. coli* Clade I, (*E. coli; E. whittamii*, *E. ruysiae* and *E. marmotae* ) with (A) sample type and (B) site. Numbers represent total number of isolates analysed per sample type or site.
**Table S5:** Odds ratios (OR) and 95% confidence intervals (CI) from binomial generalised linear mixed models (GLMMs) assessing the association between log‐transformed *E. coli* concentrations (log_10_MPN per 100 mL) and the presence of specific bacterial phylotypes (A to G) and *E. marmotae* . The binary outcome variable indicated phylotype presence (1) or absence (0). Log‐transformed *E. coli* concentration was included as a fixed effect predictor. Site was included as a random intercept to account for clustering of isolates within sampling locations. Odds ratios represent the change in odds of phylotype detection per unit increase in log_10_ *E. coli* concentration.
**Figure S3:** emi470278‐sup‐0001‐FigureS1‐S6‐TableS1‐S8.docx. *Escherichia* isolate gST relative abundance categorised at the sample type level across individual sites. For each site the relative abundance of generic *E. coli* (phylotype A to G) gSTs and Clade (non‐*E. coli Escherichia*; *E. marmotae* , *E. ruysiae* or *E. whittamii*) gSTs was determined for each sample type to compare overall gST diversity levels. Numbers represent total number of unique gSTs per site.
**Figure S4:** Sankey plot where *gnd* sequence types (gSTs) were examined at the *Escherichia* isolate level (*n* = 1347). Excluding triplet combinations of Site, Sample type and gST with a frequency of ≤ 4 resulted in the removal of 243 (92.0%) of all gSTs.
**Figure S5:** Rarefaction analysis of *Escherichia* community data. Analysis was performed across samples (*n* = 326) and generated flattened curves indicating that the amplicon sequencing depth was sufficient to accurately estimate ASV richness and reflected that the observed diversity was close to the true diversity of the respective target environmental communities.
**Figure S6:** Alpha and beta diversity of *Escherichia coli* and *E. marmotae* , *E. ruysiae* , and *E. whittamii* . Diversity metrics were calculated from metabarcoding data of *gnd* sequence types (gSTs; *n* = 416) matching *Escherichia* spp. in the *gnd* reference database (gndDb), which contains > 700 hypervariable partial *gnd* sequences from *Escherichia* species. Diversity profiles were derived from 326 environmental samples analysed by *gnd* amplicon metabarcoding. Amplicon sequence variants were identified using DADA2 and matched to gSTs in *gndDb*. For alpha diversity Chao1 richness and Shannon diversity indices are shown as boxplots, with median (line), interquartile range (box), and whiskers extending to the most extreme values within 1.5× the interquartile range. (A, B) Student's *t*‐tests comparing Chao1 and Shannon indices from Site 1 to each other site. (C, D) Student's *t*‐tests comparing Chao1 and Shannon indices from avian faeces to other environmental samples. Pairwise comparison *p* values were adjusted for multiple testing using the Benjamini–Hochberg false discovery rate (FDR < 0.05). Significance codes: *p < 0.05, **p < 0.01, ***p < 0.001, ****p < 0.0001, ns = not significant (*p* > 0.05). A one‐way ANOVA was used to compare gST (*n* = 416) read counts across all sites and sample types. For beta diversity principal component analysis (PCA) illustrates variation and spatial distribution of gSTs, showing differences by (E) land use and (F) sample type for environmental samples collected from 10 distinct sampling sites.
**Table S6A:** Estimated mean differences and 95% confidence intervals (CI) from a linear mixed‐effects model evaluating the association between microbial diversity measures and freshwater log‐transformed *E. coli* concentrations (log_10_ MPN per 100 mL). The response variable was the difference between the base‐10 logarithm of *E. coli* concentration from paired sites, forcing the analysis to reflect within‐pair differences rather than between‐pair variation. Microbial diversity was included as a continuous fixed effect, using the difference of either Shannon diversity or Chao1 richness of gSTs (matching gndDb) across paired sites as the predictor. Site type was included as a fixed effect. Estimates represent the difference in mean log_10_ *E. coli* concentration per unit increase of the diversity metric.
**Table S6B:** Estimated mean differences and 95% confidence intervals (CI) from a linear mixed‐effects model evaluating the association between site type (impacted vs. reserve) and log‐transformed *E. coli* concentrations (log_10_ MPN per 100 mL). The response variable was the base‐10 logarithm of *E. coli* concentration. Site type was included as a fixed effect, and site was included as a random intercept to account for clustering of samples within sites. Estimates represent the difference in mean log_10_ *E. coli* concentration between impacted and reserve sites, with impacted sites treated as the reference category.
**Table S6C:** Fixed effect estimates and 95% confidence intervals (CI) from a linear mixed‐effects model assessing the association between log‐transformed *E. coli* concentrations (log_10_ MPN per 100 mL) and distance (km) between impacted sites and their paired reserve sites. Only impacted sites were included in the analysis where site was included as a random intercept to account for non‐independence within reserve‐impacted site pairs. Estimates represent the change in mean log_10_ *E. coli* concentration per km increase in distance from the reserve site.
**Table S6D:** Fixed effect estimates and 95% confidence intervals (CI) from a linear mixed‐effects model evaluating the association between the total number of ruminant livestock upstream of sampling site and log‐transformed *E. coli* concentrations (log_10_ MPN per 100 mL). The response variable was the base‐10 logarithm of *E. coli* concentration. Number of ruminants/1000 was included as a continuous fixed effect predictor. Site was included as a random intercept to account for clustering of samples within sites. Estimates represent the expected change in log_10_ *E. coli* concentration per additional 100 ruminants in the catchment.
**Table S6E:** Fixed effect estimates and 95% confidence intervals (CI) from a linear mixed‐effects model evaluating the association between the total catchment area (Ha) divided by 1000 and log‐transformed *E. coli* concentrations (log_10_ MPN per 100 mL). The response variable was the base‐10 logarithm of *E. coli* concentration. Catchment area (Ha/1000) was included as a continuous fixed effect predictor. Site was included as a random intercept to account for clustering of samples within sites. Estimates represent the expected change in log_10_ *E. coli* concentration per additional 100 Ha catchment area.
**Table S6F:** Fixed effect estimates and 95% confidence intervals (CI) from linear mixed‐effects models evaluating the relationship between microbial diversity and distance from reserve sites. Models were fitted separately using either Shannon diversity or Chao1 richness from gSTs matching gndDb as the outcome variables. The explanatory variable was distance (km) between each impacted site and its paired reserve site. Only impacted sites were included in the analysis where site was included as a random intercept to account for non‐independence within reserve‐impacted site pairs. Estimates reflect the change in microbial diversity per km increase in distance from the reserve site.
**Table S7:** Odds ratios (OR) and 95% confidence intervals (CI) from binomial generalised linear mixed models assessing the association between microbial diversity and the presence of *stx*1, *stx*2, or eae genes from sample enrichments. Models were fitted using a logit link with gene presence (*C*_*q*_ < 35, 1) or absence (*C*_*q*_ > 35, 0) from individual sample enrichments as the binary outcome variable. Microbial diversity was included as a continuous fixed effect, using either Shannon diversity or Chao1 richness from metabarcoding community analysis with gSTs matching gndDb as the predictor. Site was included as a random intercept to account for clustering of isolates within sampling sites. Odds ratios reflect the change in odds of gene detection per unit increase in the diversity metric.
**Table S8:** Odds ratios (OR) and 95% confidence intervals (CI) from binomial generalised linear mixed models assessing associations between *E. coli* virulence gene detection (*stx1*, *stx2*, *eae*) and the occurrence of *E. coli* phylotypes B1, B2, and *E. marmotae* at the sample level. Each model was fitted using a logit link with phylotype presence/absence as the binary response variable, the presence of an individual virulence gene as the fixed effect, and site included as a random intercept to account for within‐site clustering. Odds ratios represent the relative odds of detecting a given phylotype in samples positive for a virulence gene compared with gene‐negative samples. Significant associations (*p* < 0.05) are shown in bold.

## Data Availability

Illumina *gnd* metabarcoding sequence data are deposited under NCBI Short Read Archive BioProject number PRJNA1262645 (BioSamples: SAMN48476962‐SAMN48477304). *gnd* reference sequences from gndDb are available from https://github.com/mEpiLab/gnd.
