# Supplementary material for: Escherichia Species Diversity Across Pristine and Impacted Catchments: Evidence for Avian Sources of Escherichia marmotae in Aotearoa/New Zealand
Source: Environ Microbiol Rep. 2026 Jan 22;18(1):e70278. doi: 10.1111/1758-2229.70278 (PMC12826111; doi:10.1111/1758-2229.70278)
Supplement: Supplementary file 1 — Figure S1: Catchment map of sampling sites. Five paired sites consisted of an upstream headwater site (odd‐numbers, shown in blue) located within a pristine native bush reserve, and a corresponding downstream site (even‐numbers, shown in orange) impacted by pastoral or urban land use. Table S1: New Zealand land‐use and livestock data details associated with each sampling site. a No water was detected in these catchments as extensive native forest cover precludes satellite detection of lower order streams. b refers to the number of livestock animals in the upstream catchment area. Table S2: New Zealand sample site details where water, soil, sediment, biofilm (periphyton) and faecal samples were obtained for this study. a Linear distance between paired sites; b number of samples where Escherichia isolated; c not collected, sandy riverbed. Table S3: Prevalence of E. coli phylotypes and non‐ E. coli Escherichia species from the 10 sample sites. The odd‐numbered sites refer to those within native bush reserves and the even‐numbered sites refer to those within impacted sites and more heavily influenced by diffuse faecal sources. Sites are separated into individual Site pairs contained within the same freshwater catchment. Table S4: Odds ratios (OR) and 95% confidence intervals (CI) from binomial generalised linear mixed models (GLMMs) assessing the association between sample type and the generic E. coli (phylotypes A to G) and non‐ E. coli Escherichia ( E. marmotae , E. ruysiae and E. whittamii ). Models were fitted using a logit link function, with the binary outcome variable indicating presence or absence of response variable bacteria from individual sample enrichments. Sample type was included as a fixed effect, and site was included as a random intercept to account for clustering. Odds ratios represent the change in odds of an isolate being specified as a particular phylotype relative to the reference avian faeces sample type. Figure S2: Relative abundance (%) of E. c [file EMI4-18-e70278-s001.docx]

**Supplementary Information.**

*Escherichia* species diversity across pristine and impacted catchments: Evidence for avian sources of *Escherichia marmotae* in Aotearoa/New Zealand

Running title: Avian sources of *Escherichia marmotae*

Adrian L. Cookson*^,1,2^, Marie Moinet^1^, Jonathan C. Marshall^2,3^, Patrick J. Biggs^2,4,5^, Lynn E. Rogers^1^, Rose M. Collis^1^, Megan Devane^6^, Rebecca Stott^7^, Richard Muirhead^8^

^1^ New Zealand Institute for Bioeconomy Science, AgResearch Group, Hopkirk Research Institute, Massey University, Palmerston North, New Zealand

^2^ ^m^EpiLab, School of Veterinary Science, Massey University, Palmerston North, New Zealand

^3^ School of Mathematics and Computational Sciences, Massey University, Palmerston North, New Zealand

^4^ School of Food Technology and Natural Sciences, Massey University, Palmerston North, New Zealand

^5^ New Zealand Food Safety Science and Research Centre, Massey University, Palmerston North, New Zealand

^6^ New Zealand Institute for Public Health and Forensic Science, Christchurch, New Zealand

^7^ Earth Sciences New Zealand, Hamilton, New Zealand

^8^ New Zealand Institute for Bioeconomy Science, AgResearch Group, Invermay Agricultural Centre, Mosgiel, New Zealand

***Corresponding author:** Adrian Cookson, Food System Integrity, Bioeconomy Science Institute, Hopkirk Research Institute, Massey University, Cnr University Avenue and Library Road, Private Bag 11008, Palmerston North, 4442, New Zealand.

Email: [adrian.cookson@agresearch.co.nz](mailto:adrian.cookson@agresearch.co.nz)

Telephone: +64 (0)6 351 8681

*Environmental sampling and bacterial recovery*

At each site: water, sediment, soil, aquatic biofilm (periphyton) (not Site03) and opportunistic avian and mammalian fecal samples were collected. *E. coli* were enumerated from water samples (100 mL) using Colilert-18 and Quanti-Tray/2000 (IDEXX, ME, USA) incubated at 35°C (18 to 21 hours) for the recovery of stressed cells to determine most probable number of *E. coli* per 100 mL (MPN per 100 mL). For the recovery of individual isolates water samples (100 mL) were filtered through 0.45μm nitrocellulose filters using positive pressure with the filters placed aseptically on CHROMagar™ ECC plates and incubated at 35°C (18 to 21 hours). Thereafter individual colonies were subcultured onto fresh CHROMagar™ ECC plates and incubated as described above. For water samples with low *E. coli* MPN per 100 mL concentrations, filters were enriched in 10 mL EC broth incubated at 35°C (18 to 21 hours) with 10 µL culture streaked onto CHROMagar™ ECC.

Freshwater sediment samples were obtained using a stainless-steel shovel and sieved through a mesh size of approximately 3 mm to retain coarse particles. Soil samples were taken from locations 5 to 10 m away from the freshwater corresponding freshwater sample sites. A composite soil sample from each site (~70 g) was obtained using a sterile 150 mm stainless steel corer. Sediment and soil material (1 g) was enriched in 9 mL EC broth by incubating at 35°C (18 to 21 hours) followed by streaking 5 µL enrichment onto CHROMagar™ ECC plates.

Biofilm samples were obtained from all stream sites by carefully removing a fully submerged rock from the waterway and wiping an area of approximately 100 cm^2^ using a sterile sponge swab (EZ-Reach Sponge Sampler, World Bioproducts, Washington, USA). The sterile sponge swab was stomached for 1 min with 25 mL EC broth (Oxoid, Hampshire, UK) and incubated at 35°C (18 to 21 hours). Broth culture (50 μl) was inoculated onto CHROMagar™ ECC plates, streaked for individual colonies and incubated at 35°C (18 to 21 hours).

Opportunistic fecal material was obtained using a sterile Amies swab (Copan Diagnostics Inc., Brescia, Italy) or sterile specimen container with scoop cap, diluted 1:100 in EC broth and incubated on CHROMagar™ ECC plates for isolation of individual colonies as before.

For all sample types, four distinct sub-cultured colonies per sample preparation were resuspended in EC broth containing glycerol (33% [w/v]) and stored at -80°C. Washed boiled DNA lysates for use as template DNA in subsequent PCRs were made from overnight cultures or from a representative colony from an isolation plate. Briefly 1 mL of culture was centrifuged at 13,000 x g and the supernatant removed. The pellet was resuspended in 0.01 M phosphate buffered saline (PBS, pH 7.4) and centrifuged again. The supernatant was removed once more, and the pellet resuspended in 1 mL molecular biology grade water. For DNA preparations from sub-cultured bacterial isolates, one or two well-spaced colonies were removed from culture agar plates using a sterile loop and emulsified in 400 µl molecular biology grade water. Bacteria were lysed by heating the resuspended bacteria at 100°C for 10 min and then stored at -20°C.


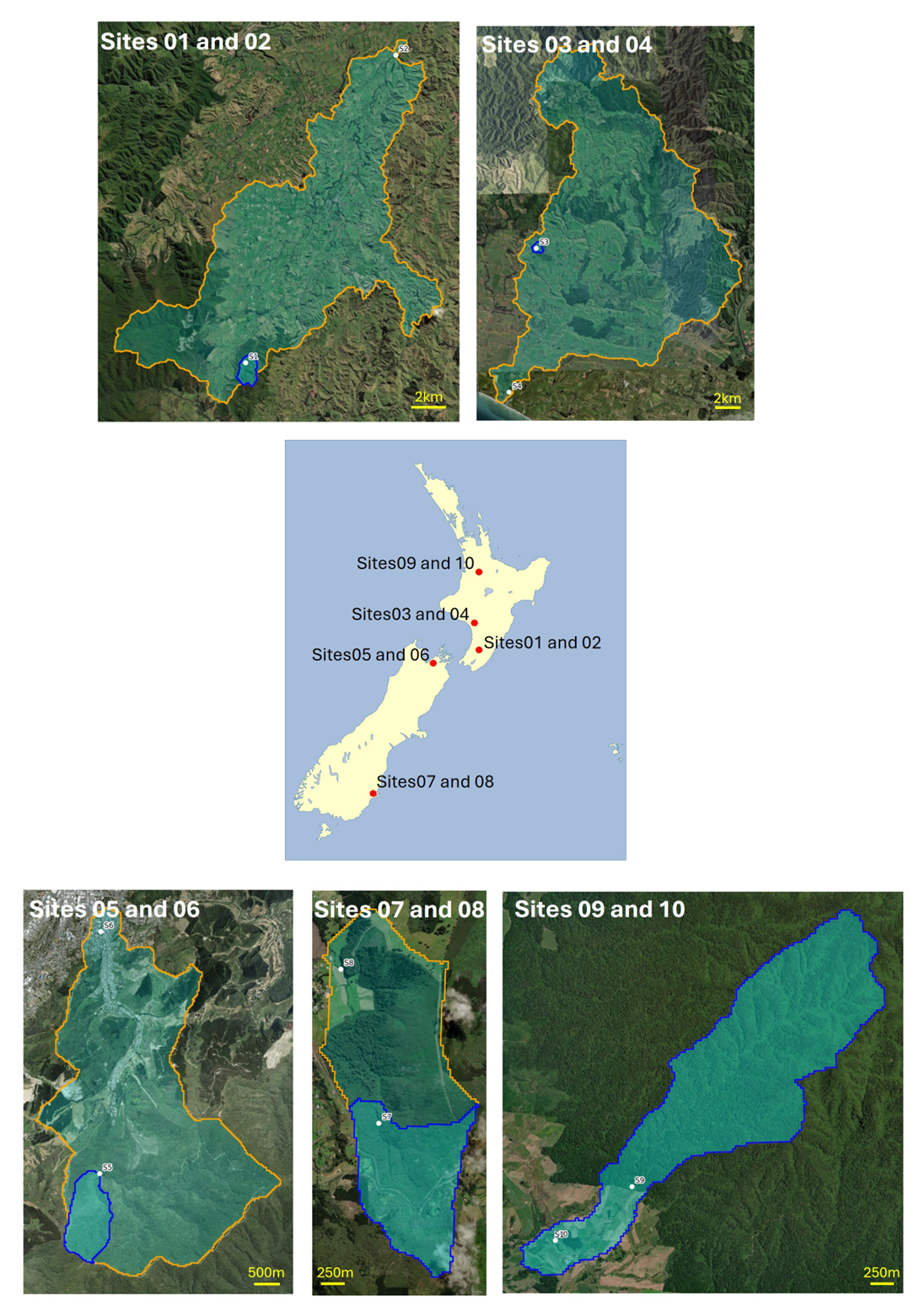


**Supplementary Figure S1.** Catchment map of sampling sites. Five paired sites consisted of an upstream headwater site (odd-numbers, shown in blue) located within a pristine native bush reserve, and a corresponding downstream site (even-numbers, shown in orange) impacted by pastoral or urban land use.

**Supplementary Table S1.** New Zealand land-use and livestock data details associated with each sampling site. ^a^ No water was detected in these catchments as extensive native forest cover precludes satellite detection of lower order streams. ^b^ refers to the number of livestock animals in the upstream catchment area.

| ***Site id*** | ***Total area, Ha*** | ***bare, Ha (%)*** | ***built, Ha (%)*** | ***crops, Ha (%)*** | ***grass, Ha (%)*** | ***shrub and scrub, Ha (%)*** | ***trees, Ha (%)*** | ***water, Ha (%)*** | ***Catchment length (km)*** | ***Dairy (n) u/s*** ^b^ | ***Beef (n) u/s*** ^b^ | ***Sheep (n) u/s*** ^b^ |
| --- | --- | --- | --- | --- | --- | --- | --- | --- | --- | --- | --- | --- |
| Site01 | 176.03 | 0 | 0 | 0 | 0 | 0 | 100 | 0 ^a^ | 1.36 | 0 | 0 | 0 |
| Site02 | 16412.68 | 0.36 | 0.28 | 0 | 77.96 | 3.25 | 18.14 | 0.01 | 40.57 | 7735 | 4173 | 34114 |
| Site03 | 38.89 | 0 | 0 | 0 | 14.01 | 0 | 85.99 | 0 ^a^ | 0.7 | 0 | 6 | 29 |
| Site04 | 19079.92 | 2.29 | 0.08 | 0.45 | 47.24 | 8.14 | 41.8 | 0.01 | 52.1 | 75 | 8643 | 59809 |
| Site05 | 110.99 | 0 | 0 | 0 | 0 | 28.76 | 71.24 | 0 ^a^ | 1.73 | 0 | 0 | 0 |
| Site06 | 1718.24 | 1.61 | 5.1 | 0 | 7.16 | 13.07 | 73.06 | 0 ^a^ | 9.0 | 0 | 77 | 528 |
| Site07 | 174.31 | 0 | 0 | 0 | 8.1 | 67.17 | 24.73 | 0 ^a^ | 0.7 | 0 | 3 | 62 |
| Site08 | 454.64 | 0 | 0 | 0 | 13.56 | 50.22 | 36.15 | 0.07 | 3.03 | 0 | 22 | 521 |
| Site09 | 384.72 | 0 | 0 | 0 | 0 | 0 | 100 | 0 ^a^ | 3.6 | 0 | 0 | 0 |
| Site10 | 440.63 | 0 | 0 | 0 | 12.69 | 0 | 87.31 | 0 ^a^ | 4.9 | 51 | 20 | 45 |

**Supplementary Table S2.** New Zealand sample site details where water, soil, sediment, biofilm (periphyton) and fecal samples were obtained for this study. ^a^ Linear distance between paired sites; ^b^ number of samples where *Escherichia* isolated; ^c^ not collected, sandy riverbed

| ***Site*** | ***Name (linear distance, km***^a^***)*** | ***GPS*** | ***Altitude (m)*** | ***Land-use*** | ***E. coli MPN per 100ml water*** | **Samples (n)**^b^ | | |  | | |
| --- | --- | --- | --- | --- | --- | --- | --- | --- | --- | --- | --- |
|  |  |  |  |  |  | ***water*** | ***sediment*** | ***soil*** | ***biofilm*** | ***avian feces*** | ***mammalian feces*** |
| Site01 | Pūkaha Mount Bruce (20.9km) | -40.7341, 175.6377 | 341 | native forest | geomean 20 (min 4; max 133 | 6 (3) | 6 (3) | 6 (0) | 6 (4) | 17 (10) | 0 |
| Site02 | Hamua Bridge (20.9km) | -40.5654, 175.7461 | 171 | dairy, sheep, beef farming | geomean 2624 (min 272; max 8130) | 6 (6) | 4 (4) | 6 (6) | 5 (5) | 3 (3) | 9 (9) |
| Site03 | Bushy Park (9.6km) | -39.7943, 174.9241 | 239 | native forest | geomean 50.0 (min 1; max 275.5) | 6 (6) | 6 (5) | 6 (3) | 0^c^ | 22 (16) | 0 |
| Site04 | Kai Iwi (9.6km) | -39.8788, 174.9031 | 18 | dairy, sheep, beef farming | geomean 443 (min 261.3; max 2419.6) | 6 (6) | 6 (6) | 6 (6) | 6 (6) | 8 (8) | 3 (3) |
| Site05 | Brook Waimarama (4.6km) | -41.3206, 173.2891 | 175 | native forest | geomean 2.9 (min <1.0; max 36.9) | 6 (3) | 6 (3) | 6 (4) | 6 (5) | 21(16) | 0 |
| Site06 | Manuka St (4.6km) | -41.2788, 173.2894 | 17 | urban, sheep, beef farming | geomean 177.6 (min 37.3; max 1732.9) | 6 (6) | 6 (6) | 6 (5) | 6 (6) | 21 (20) | 1 (1) |
| Site07 | Orokonui Ecosanctuary (1.82km) | -45.7717, 170.5936 | 137 | native forest | geomean 2.4 (min <1.0; max 33.6) | 6 (6) | 6 (6) | 6 (1) | 6 (6) | 24 (23) | 0 |
| Site08 | Orokonui Creek (1.82km) | -45.7559, 170.5880 | 10 | sheep, beef farming | geomean 75.6 (min 4.1; max 727.0) | 6 (6) | 6 (6) | 6 (5) | 6 (6) | 21 (19) | 0 |
| Site09 | Maungatautari (1.01km) | -38.0448, 175.5478 | 306 | native forest | geomean 2.1 (min <1.0; max 14.5) | 6 (4) | 6 (5) | 6 (2) | 6 (6) | 16 (11) | 0 |
| Site10 | Maru Rd (1.01km) | -38.0500, 175.5384 | 239 | dairy, sheep, beef farming | geomean 62.7 (min 6.3; max 613.1) | 6 (6) | 6 (6) | 6 (6) | 6 (6) | 9 (7) | 7 (6) |
|  |  |  |  |  | **TOTAL** | 60 (52) | 58 (50) | 60 (38) | 53 (50) | 162 (133) | 20 (19) |

**Supplementary Table S3.** Prevalence of *E. coli* phylotypes and non-*E. coli Escherichia* species from the ten sample sites. The odd-numbered sites refer to those within native bush reserves and the even-numbered sites refer to those within impacted sites and more heavily influenced by diffuse fecal sources. Sites are separated into individual Site pairs contained within the same freshwater catchment.

| **Site** | **Phylotype or cryptic clade** | | | | | | | | | **Non-*E. coli Escherichia* species** | | | |
| --- | --- | --- | --- | --- | --- | --- | --- | --- | --- | --- | --- | --- | --- |
|  | **A** | **B1** | **B2** | **C** | **D** | **E** | **F** | **G** | **Clade I** | ***E. whittamii*** | ***E. ruysiae*** | ***E. marmotae*** | **Untyped** |
| **Site01** | 0.00 | 0.17 | 0.24 | 0.00 | 0.10 | 0.02 | 0.02 | 0.00 | 0.00 | 0.00 | 0.00 | 0.17 | 0.00 |
| **Site02** | 0.12 | 1.00 | 0.12 | 0.15 | 0.03 | 0.27 | 0.00 | 0.00 | 0.00 | 0.00 | 0.00 | 0.06 | 0.00 |
| **Site03** | 0.03 | 0.43 | 0.23 | 0.00 | 0.25 | 0.05 | 0.00 | 0.00 | 0.00 | 0.00 | 0.00 | 0.20 | 0.03 |
| **Site04** | 0.03 | 0.83 | 0.33 | 0.17 | 0.31 | 0.19 | 0.00 | 0.08 | 0.03 | 0.00 | 0.00 | 0.08 | 0.03 |
| **Site05** | 0.02 | 0.04 | 0.36 | 0.00 | 0.02 | 0.02 | 0.04 | 0.07 | 0.00 | 0.00 | 0.00 | 0.42 | 0.00 |
| **Site06** | 0.11 | 0.61 | 0.28 | 0.09 | 0.04 | 0.15 | 0.02 | 0.02 | 0.00 | 0.00 | 0.00 | 0.48 | 0.00 |
| **Site07** | 0.06 | 0.15 | 0.25 | 0.02 | 0.02 | 0.02 | 0.00 | 0.02 | 0.00 | 0.00 | 0.06 | 0.65 | 0.00 |
| **Site08** | 0.11 | 0.76 | 0.16 | 0.13 | 0.16 | 0.20 | 0.02 | 0.11 | 0.00 | 0.00 | 0.00 | 0.27 | 0.00 |
| **Site09** | 0.00 | 0.20 | 0.35 | 0.00 | 0.23 | 0.18 | 0.03 | 0.03 | 0.00 | 0.00 | 0.00 | 0.25 | 0.00 |
| **Site10** | 0.03 | 0.65 | 0.43 | 0.03 | 0.20 | 0.18 | 0.05 | 0.03 | 0.00 | 0.03 | 0.00 | 0.13 | 0.00 |

**Supplementary Table S4.** Odds ratios (OR) and 95% confidence intervals (CI) from binomial generalized linear mixed models (GLMMs) assessing the association between sample type and the generic *E. coli* (phylotypes A to G) and non-*E. coli Escherichia* (*E. marmotae*, *E. ruysiae* and *E. whittamii*). Models were fitted using a logit link function, with the binary outcome variable indicating presence or absence of response variable bacteria from individual sample enrichments. Sample type was included as a fixed effect, and site was included as a random intercept to account for clustering. Odds ratios represent the change in odds of an isolate being specified as a particular phylotype relative to the reference avian feces sample type.

| **Generic *E. coli*** | **Fixed effects** | **OR** | **95% CI** | **z value** | **P value** |
| --- | --- | --- | --- | --- | --- |
|  | **Avian feces (reference)** | 1 | NA | NA | NA |
|  | Biofilm | 4.432 | 1.743 - 11.27 | 3.128 | **0.002** |
|  | Mammalian feces | 2.458 | 0.298 - 20.29 | 0.835 | 0.404 |
|  | Sediment | 2.332 | 1.066 - 5.103 | 2.119 | **0.034** |
|  | Soil | 0.417 | 0.204 - 0.854 | -2.391 | **0.017** |
|  | Water | 4.15 | 1.729 - 9.96 | 3.186 | **0.001** |
|  |  |  |  |  |  |
| **Non-*E. coli* *Escherichia* spp.** | **Fixed effects** | **OR** | **95% CI** | **z value** | **P value** |
|  | **Avian feces (reference)** | 1 | NA | NA | NA |
|  | Biofilm | 0.521 | 0.243 - 1.12 | -1.675 | 0.094 |
|  | Mammalian feces | 0.326 | 0.067 - 1.59 | -1.387 | 0.166 |
|  | Sediment | 0.427 | 0.199 - 0.916 | -2.185 | **0.029** |
|  | Soil | 0.471 | 0.224 - 0.993 | -1.978 | **0.048** |
|  | Water | 1.028 | 0.522 - 2.024 | 0.079 | 0.9372 |

A


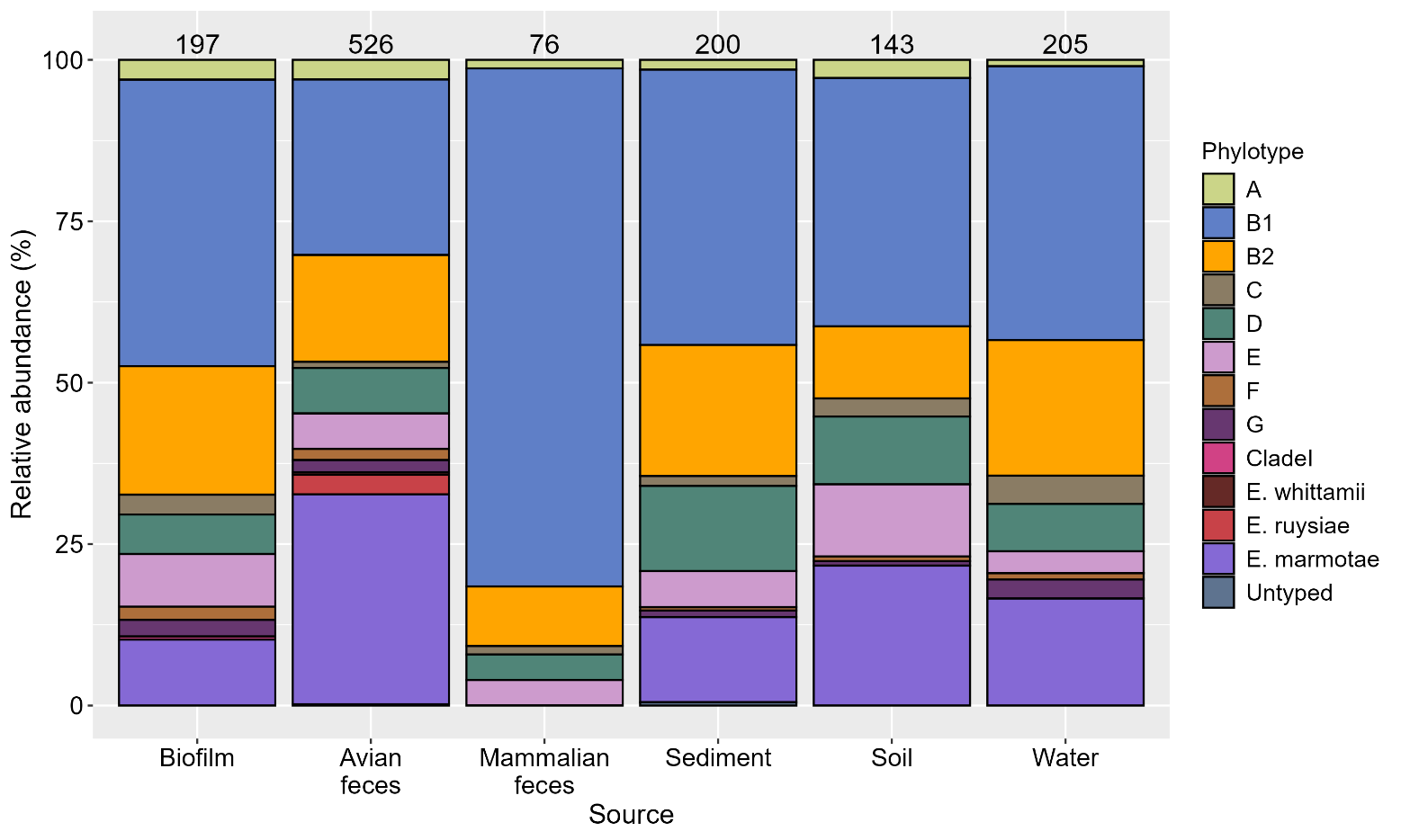


B


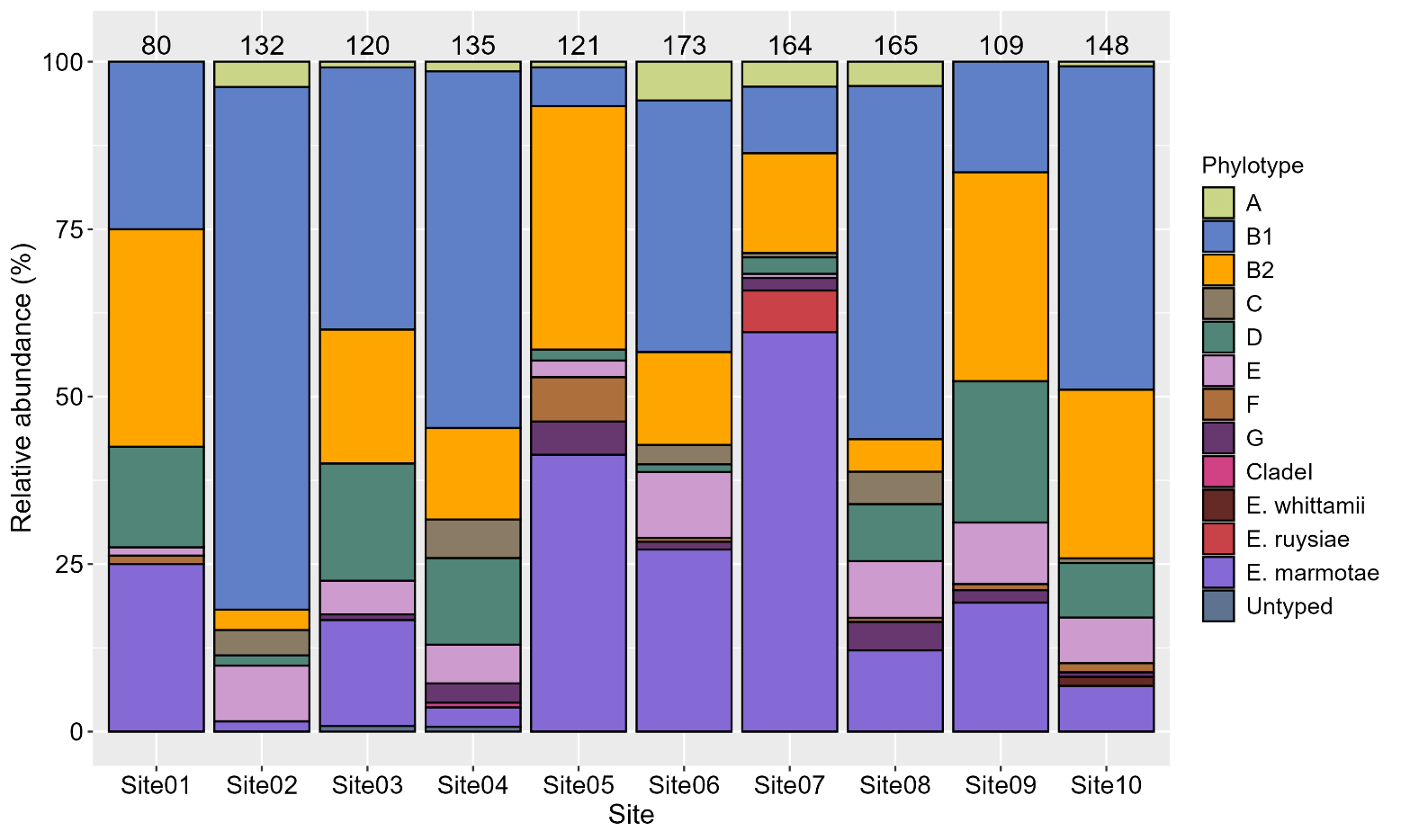


**Supplementary Figure S2.** Relative abundance (%) of *E. coli* phylotypes (A to G) and *E. coli* Clade I, *E. coli;* *E. whittamii, E. ruysiae* and *E. marmotae*) with (A) sample type and (B) site. Numbers represent total number of isolates analysed per sample type or site.

**Supplementary Table S5.** Odds ratios (OR) and 95% confidence intervals (CI) from binomial generalized linear mixed models (GLMMs) assessing the association between log-transformed *E. coli* concentrations (log_10_MPN per 100 mL) and the presence of specific bacterial phylotypes (A to G) and *E. marmotae*. The binary outcome variable indicated phylotype presence (1) or absence (0). Log-transformed *E. coli* concentration was included as a fixed effect predictor. Site was included as a random intercept to account for clustering of isolates within sampling locations. Odds ratios represent the change in odds of phylotype detection per unit increase in log_10_ *E. coli* concentration.

|  | **Fixed effects** | **OR** | **95% CI** | **z value** | **P value** |
| --- | --- | --- | --- | --- | --- |
|  | Phylotype negative (reference) | 1 | ref |  |  |
| Phylotype A | log_10_ MPN | 2.59 | 0.68 – 9.82 | 1.40 | 0.161 |
| Phylotype B1 | log_10_ MPN | 3.34 | 1.67 - 6.71 | 3.396 | **0.0006** |
| Phylotype B2 | log_10_ MPN | 1.22 | 0.85 - 1.74 | 1.087 | 0.277 |
| Phylotype C | log_10_ MPN | 1.31 | 0.79 - 2.19 | 1.034 | 0.301 |
| Phylotype D | log_10_ MPN | 0.94 | 0.57 - 1.55 | -0.248 | 0.804 |
| Phylotype E | log_10_ MPN | 1.13 | 0.66 - 1.95 | 0.44 | 0.66 |
| Phylotype F | log_10_ MPN | 0.98 | 0.38 - 2.53 | -0.053 | 0.958 |
| Phylotype G | log_10_ MPN | 1.3 | 0.58 - 2.93 | 0.633 | 0.527 |
| *E. marmotae* | log_10_ MPN | 1.13 | 0.62 - 2.03 | 0.397 | 0.691 |


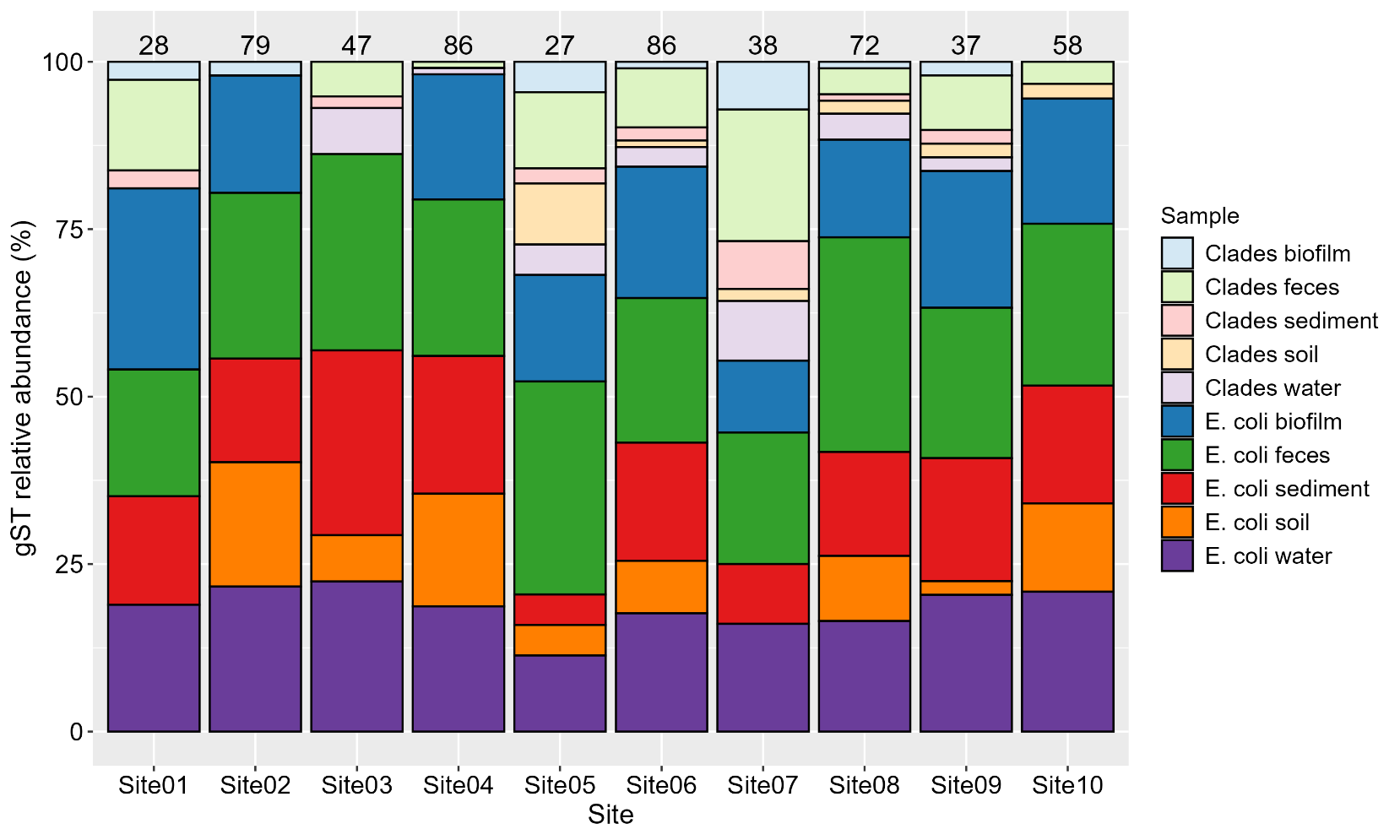


**Supplementary Figure S3.** *Escherichia* isolate gST relative abundance categorised at the sample type level across individual sites. For each site the relative abundance of generic *E. coli* (phylotype A to G) gSTs and Clade (non-*E. coli Escherichia*; *E. marmotae*, *E. ruysiae* or *E. whittamii*) gSTs was determined for each sample type to compare overall gST diversity levels. Numbers represent total number of unique gSTs per site.


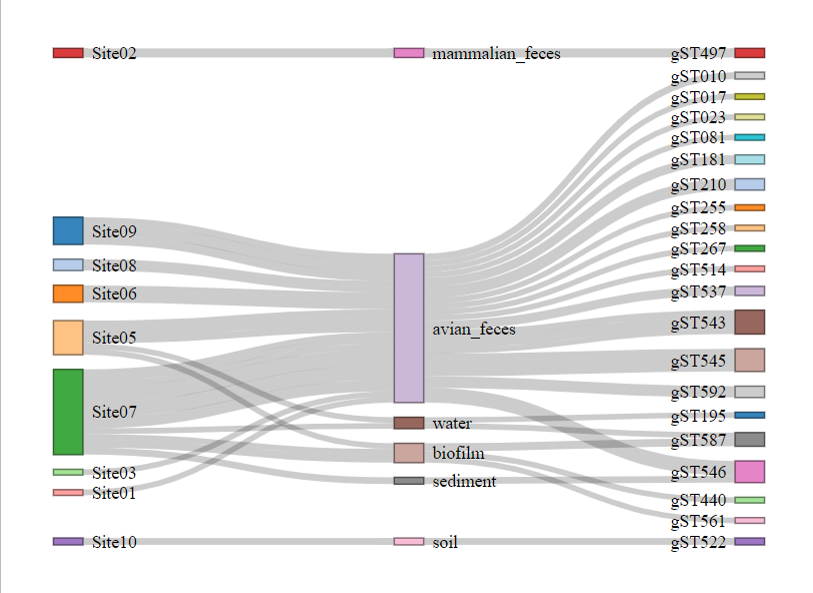


**Supplementary Figure S4.** Sankey plot where *gnd* sequence types (gSTs) were examined at the *Escherichia* isolate level (n=1347). Excluding triplet combinations of Site, Sample type and gST with a frequency of ≤ four resulted in the removal of 243 (92.0%) of all gSTs.


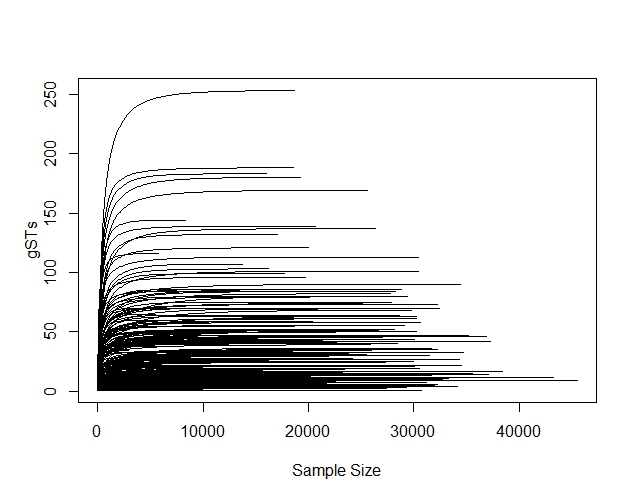


**Supplementary Figure S5.** Rarefaction analysis of *Escherichia* community data. Analysis was performed across samples (n=326) and generated flattened curves indicating that the amplicon sequencing depth was sufficient to accurately estimate ASV richness and reflected that the observed diversity was close to the true diversity of the respective target environmental communities.


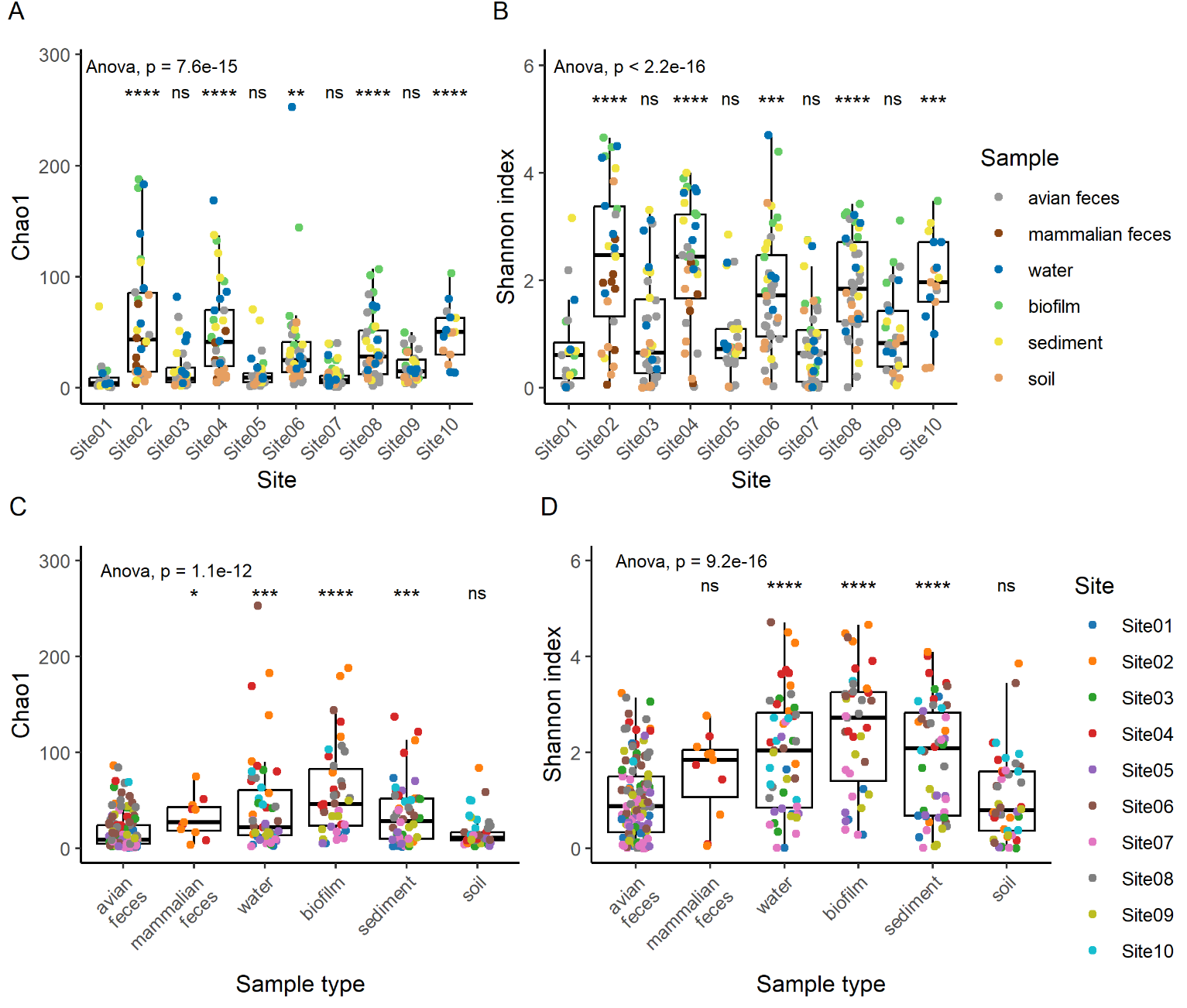


E

**
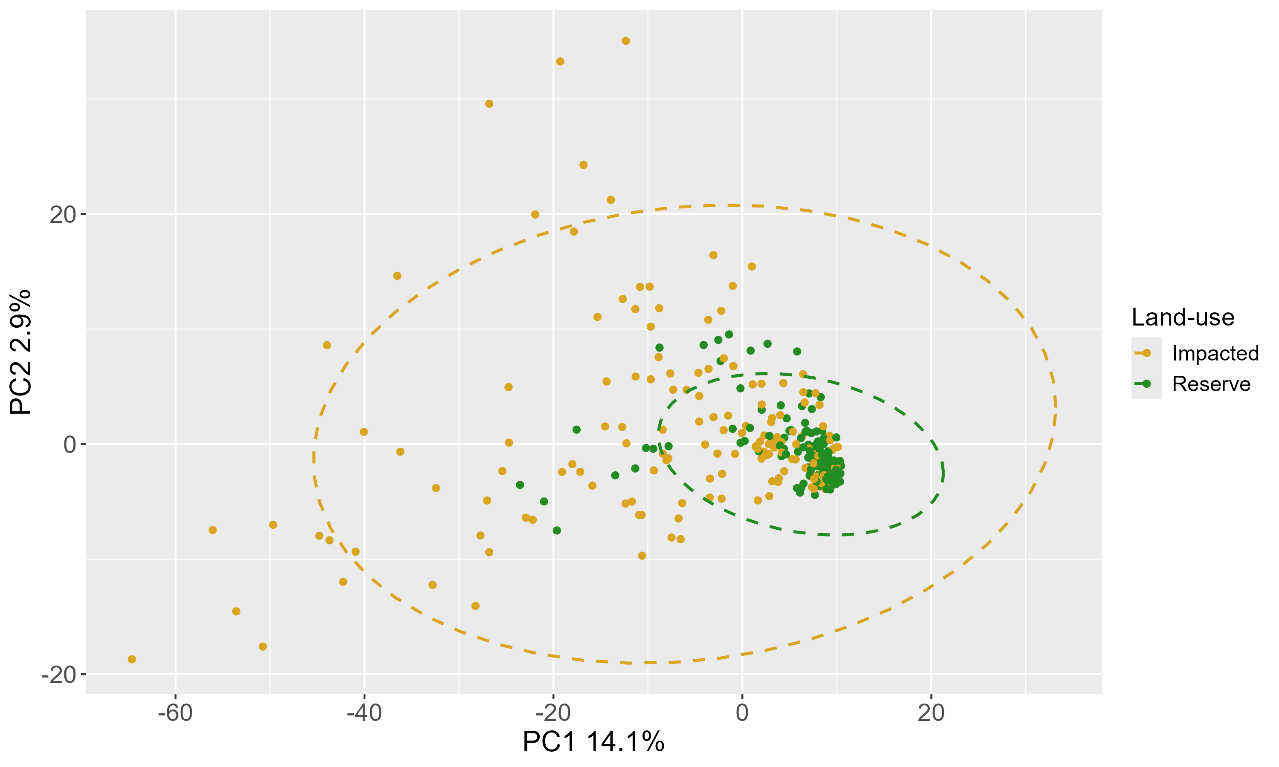
**

F

**
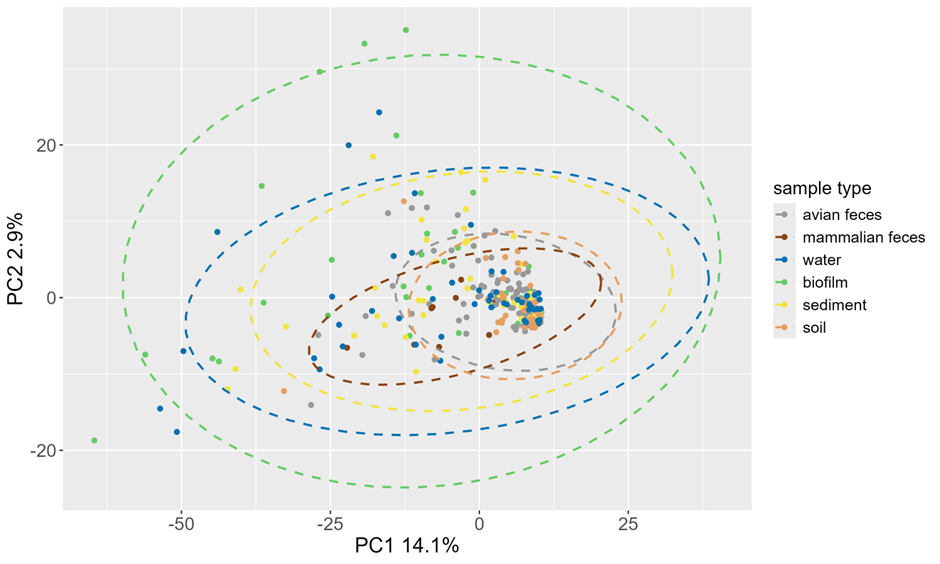
**

**Supplementary Figure S6.** Alpha and beta diversity of *Escherichia coli* and *E. marmotae*, *E. ruysiae*, and *E. whittamii*. Diversity metrics were calculated from metabarcoding data of *gnd* sequence types (gSTs; n=416) matching *Escherichia* spp. in the *gnd* reference database (gndDb), which contains >700 hypervariable partial *gnd* sequences from *Escherichia* species. Diversity profiles were derived from 326 environmental samples analysed by *gnd* amplicon metabarcoding. Amplicon sequence variants were identified using DADA2 and matched to gSTs in *gndDb*. For alpha diversity Chao1 richness and Shannon diversity indices are shown as boxplots, with median (line), interquartile range (box), and whiskers extending to the most extreme values within 1.5× the interquartile range. Figures (A) and (B): Student’s *t*-tests comparing Chao1 and Shannon indices from Site 1 to each other site. Figures (C) and (D): Student’s *t*-tests comparing Chao1 and Shannon indices from avian feces to other environmental samples. Pairwise comparison p-values were adjusted for multiple testing using the Benjamini–Hochberg false discovery rate (FDR < 0.05). Significance codes: p < 0.05 (*), p < 0.01 (**), p < 0.001 (***), p < 0.0001 (****), ns = not significant (p > 0.05). A one-way ANOVA was used to compare gST (n = 416) read counts across all sites and sample types. For beta diversity principal component analysis (PCA) illustrates variation and spatial distribution of gSTs, showing differences by (E) land use and (F) sample type for environmental samples collected from ten distinct sampling sites.

**Supplementary Table S6A.** Estimated mean differences and 95% confidence intervals (CI) from a linear mixed-effects model evaluating the association between microbial diversity measures and freshwater log-transformed *E. coli* concentrations (log₁₀ MPN per 100 mL). The response variable was the difference between the base-10 logarithm of *E. coli* concentration from paired sites, forcing the analysis to reflect within-pair differences rather than between-pair variation. Microbial diversity was included as a continuous fixed effect, using the difference of either Shannon diversity or Chao1 richness of gSTs (matching gndDb) across paired sites as the predictor. Site type was included as a fixed effect. Estimates represent the difference in mean log₁₀ *E. coli* concentration per unit increase of the diversity metric.

|  | **Fixed effect** | **Est** | **95% CI** | **t value** | **P value** |
| --- | --- | --- | --- | --- | --- |
| log_10_ MPN | Chao_gSTs | 0.007 | -0.0003 – 0.016 | 1.85 | 0.081 |
|  | Shannon_gSTs | 0.368 | 0.127 – 0.657 | 2.95 | **0.009** |

**Supplementary Table S6B.** Estimated mean differences and 95% confidence intervals (CI) from a linear mixed-effects model evaluating the association between site type (impacted vs reserve) and log-transformed *E. coli* concentrations (log_10_ MPN per 100 mL). The response variable was the base-10 logarithm of *E. coli* concentration. Site type was included as a fixed effect, and site was included as a random intercept to account for clustering of samples within sites. Estimates represent the difference in mean log_10_ *E. coli* concentration between impacted and reserve sites, with impacted sites treated as the reference category.

|  | **Land-use** | **Est** | **95% CI** | **t value** | **P value** |
| --- | --- | --- | --- | --- | --- |
| log_10_ MPN | Impacted (reference) | 1 | ref |  |  |
|  | Reserve | -2.11 | -2.87 – -1.34 | -5.36 | **0.0007** |

**Supplementary Table S6C.** Fixed effect estimates and 95% confidence intervals (CI) from a linear mixed-effects model assessing the association between log-transformed *E. coli* concentrations (log_10_ MPN per 100 mL) and distance (km) between impacted sites and their paired reserve sites. Only impacted sites were included in the analysis where site was included as a random intercept to account for non-independence within reserve-impacted site pairs. Estimates represent the change in mean log_10_ *E. coli* concentration per km increase in distance from the reserve site.

|  | **Land-use** | **Est** | **95% CI** | **t value** | **P value** |
| --- | --- | --- | --- | --- | --- |
| log_10_ MPN | Distance | 0.062 | 0.024 – 0.099 | 3.23 | **0.003** |
|  | only 'impacted' sites |  |  |  |  |

**Supplementary Table S6D.** Fixed effect estimates and 95% confidence intervals (CI) from a linear mixed-effects model evaluating the association between the total number of ruminant livestock upstream of sampling site and log-transformed *E. coli* concentrations (log_10_ MPN per 100 mL). The response variable was the base-10 logarithm of *E. coli* concentration. Number of ruminants/1000 was included as a continuous fixed effect predictor. Site was included as a random intercept to account for clustering of samples within sites. Estimates represent the expected change in log_10_ *E. coli* concentration per additional 100 ruminants in the catchment.

|  | **Fixed effects** | **Est** | **95% CI** | **t value** | **P value** |
| --- | --- | --- | --- | --- | --- |
| log_10_ MPN | Livestock (divided by 1000) | 0.033 | 0.006 - 0.059 | 2.34 | 0.047 |

**Supplementary Table S6E.** Fixed effect estimates and 95% confidence intervals (CI) from a linear mixed-effects model evaluating the association between the total catchment area (Ha) divided by 1000 and log-transformed *E. coli* concentrations (log_10_ MPN per 100 mL). The response variable was the base-10 logarithm of *E. coli* concentration. Catchment area (Ha/1000) was included as a continuous fixed effect predictor. Site was included as a random intercept to account for clustering of samples within sites. Estimates represent the expected change in log_10_ *E. coli* concentration per additional 100 Ha catchment area.

|  | **Fixed effect** | **Est** | **95% CI** | **t value** | **P value** |
| --- | --- | --- | --- | --- | --- |
| log_10_ MPN | Catchment area (Ha divided by 1000) | 0.041 | -0.001 - 0.085 | 1.86 | 0.100 |

**Supplementary Table S6F.** Fixed effect estimates and 95% confidence intervals (CI) from linear mixed-effects models evaluating the relationship between microbial diversity and distance from reserve sites. Models were fitted separately using either Shannon diversity or Chao1 richness from gSTs matching gndDb as the outcome variables. The explanatory variable was distance (km) between each impacted site and its paired reserve site. Only impacted sites were included in the analysis where site was included as a random intercept to account for non-independence within reserve-impacted site pairs. Estimates reflect the change in microbial diversity per km increase in distance from the reserve site.

|  | **Fixed effect** | **Est** | **95% CI** | **t value** | **P value** |
| --- | --- | --- | --- | --- | --- |
| Shannon_gSTs | Distance (km) | 0.067 | 0.016 – 0.12 | 2.62 | **0.014** |
|  | only 'impacted' sites |  |  |  |  |
| Chao1_gSTs | Distance (km) | 2.46 | 0.027 – 4.63 | 2.20 | **0.037** |
|  | only 'impacted' sites |  |  |  |  |

**Supplementary Table S7.** Odds ratios (OR) and 95% confidence intervals (CI) from binomial generalized linear mixed models assessing the association between microbial diversity and the presence of *stx*1, *stx*2, or *eae* genes from sample enrichments. Models were fitted using a logit link with gene presence (C_q_ <35, 1) or absence (C_q_ >35, 0) from individual sample enrichments as the binary outcome variable. Microbial diversity was included as a continuous fixed effect, using either Shannon diversity or Chao1 richness from metabarcoding community analysis with gSTs matching gndDb as the predictor. Site was included as a random intercept to account for clustering of isolates within sampling sites. Odds ratios reflect the change in odds of gene detection per unit increase in the diversity metric.

| **Virulence gene** | **Fixed effect** | **OR** | **95% CI** | **z value** | **P value** |
| --- | --- | --- | --- | --- | --- |
|  | sample negative (reference) | 1 | ref |  |  |
| *stx*1 | Shannon_gSTs | 2.835 | 1.796 - 4.474 | 4.476 | **<0.0001** |
| *stx*2 | Shannon_gSTs | 1.726 | 1.338 - 2.228 | 4.196 | **0.0001** |
| *eae* | Shannon_gSTs | 5.408 | 3.698 - 7.909 | 8.704 | **<0.0001** |
|  | sample negative (reference) |  |  |  |  |
| *stx*1 | Chao_gSTs | 1.038 | 1.022 - 1.054 | 4.714 | **<0.0001** |
| *stx*2 | Chao_gSTs | 1.029 | 1.017 - 1.041 | 4.874 | **<0.0001** |
| *eae* | Chao_gSTs | 1.121 | 1.089 - 1.155 | 7.611 | **<0.0001** |

**Supplementary Table S8.** Odds ratios (OR) and 95% confidence intervals (CI) from binomial generalized linear mixed models assessing associations between E. coli virulence gene detection (stx1, stx2, eae) and the occurrence of E. coli phylotypes B1, B2, and E. marmotae at the sample level. Each model was fitted using a logit link with phylotype presence/absence as the binary response variable, the presence of an individual virulence gene as the fixed effect, and site included as a random intercept to account for within-site clustering. Odds ratios represent the relative odds of detecting a given phylotype in samples positive for a virulence gene compared with gene-negative samples. Significant associations (p < 0.05) are shown in bold.

| **Phylotype** | **Fixed effect** | **OR** | **95% CI** | **z value** | **P value** |
| --- | --- | --- | --- | --- | --- |
|  | Sample negative (reference) | 1 | ref |  |  |
| B1 | *stx*1 | 17.34 | 1.97 – 152.4 | 2.57 | **0.010** |
|  | *stx*2 | 1.45 | 0.80 – 2.63 | 1.22 | 0.222 |
|  | *eae* | 5.34 | 3.0 – 9.53 | 5.68 | **<0.0001** |
| B2 | *stx*1 | 1.35 | 0.57 – 3.20 | 0.675 | 0.5 |
|  | *stx*2 | 0.52 | 0.291 - 0.92 | -2.243 | **0.025** |
|  | *eae* | 1.56 | 0.92 – 2.64 | 1.66 | 0.097 |
| *E. marmotae* | *stx*1 | 0.14 | 0.019 – 1.09 | -1.876 | 0.061 |
|  | *stx*2 | 0.89 | 0.489 – 1.62 | -3.379 | 0.705 |
|  | *eae* | 1.27 | 0.735 - 2.203 | 0.861 | 0.389 |
